# Impact of head orientation and head movement in traditional manual diagnostics of benign paroxysmal positional vertigo: a randomized controlled crossover study

**DOI:** 10.3389/fneur.2025.1654404

**Published:** 2025-10-03

**Authors:** Malene Hentze, Dan Dupont Hougaard, Herman Kingma

**Affiliations:** ^1^Balance and Dizziness Centre, Department of Otorhinolaryngology, Head and Neck Surgery and Audiology, Aalborg University Hospital, Aalborg, Denmark; ^2^Department of Clinical Medicine, Aalborg University, Aalborg, Denmark

**Keywords:** benign paroxysmal positional vertigo, vertigo, diagnostics, supine-roll test, dix-Hallpike test, TRV-chair, mechanical rotation chair, reposition chair

## Abstract

**Background:**

Tradititional manual diagnostics of Benign Paroxysmal Positional Vertigo (BPPV) include Supine Roll test (SRT) and Dix-Hallpike test (DHT). However, the influence of head orientation and -movement on the diagnostic performance remains unclear.

**Objective:**

To assess how head orientation and -movement affect the diagnostic performance of the manual SRT and DHT.

**Method:**

This prospective, randomized, crossover study was conducted at a tertiary university hospital outpatient clinic. Participants with suspected BPPV (*n* = 198) underwent (in random order) both manual and mechanical rotation chair (MRC)-based (gold standard) SRT and DHT. BPPV diagnosis required characteristic positional nystagmus. Participants were grouped as: (1) true positives (manual and MRC diagnostics detection the same BPPV nystagmus) and (2) false negatives (manual: negative, MRC: positive). Primary outcome was difference in head orientation and -movement between groups. Secondary outcome was minimal head orientation required for BPPV nystagmus detection in the manual tests.

**Results:**

With manual SRT, yaw head angles were substantially below the 90° target [right: 70.3° (95% CI: 68.7, 71.9); left: −66.2° (95% CI: −67.7, −64.6)]. Manual SRT missed a large proportion of BPPV (right: 63.3%; left: 62.5%). A minimum yaw angle of approximately ±55° appeared necessary for BPPV nystagmus detection. For the pitch angle, overshooting the −60° target (to −75°) seemed more effective than undershooting. For manual DHT, yaw angles were closer to target ±45°, though left DHT was less accurate [right: 47.4° (95% CI: 46.2, 48.7); left: −33.3° (95% CI: −34.6, −31,9)]. BPPV detection rates were higher (right: 73.2%; left: 65.9%), with a tendency toward better outcome when yaw head angle was overshot, and pitch angle ranged from −100° to −120°. Head movements varied narrowly, making it challenging to determine minimal values. No differences in head movements were found between true positive and false negative groups.

**Conclusion:**

Manual DHT effectively detected posterior BPPV. In contrast, manual SRT (without truncal rotation), lacking sufficient yaw rotation, missed most lateral BPPV. Therefore, we recommend performing manual SRT with full-body rotation or upper trunk rotation. Future research is encouraged to define optimal head orientation and -movement in BPPV diagnostics.

**Clinical trial registration:**

ClinicalTrials.gov, identifier, NCT05846711.

## 1 Introduction

Benign paroxysmal positional vertigo (BPPV) is a disease of the inner ear characterized by sudden, intense episodes of vertigo provoked by specific changes in the head orientation relative to gravity. These episodes are generally observed with latency, limited duration, and fatiguability (1, 2). BPPV has an accumulated lifetime incidence of 10% (before the age of 80) (3), making it the leading cause of vestibular vertigo at current (3–5). The one-year prevalence of BPPV attacks increases significantly with advancing age, from 0.5% in 18–39-year-olds to 3.4% in those over 60 years (3). BPPV is more common in women (female-to-male ratio of 1.5–2.3) (3, 4, 6) and its appearance is associated with head trauma and concurrent inner ear conditions such as infections, surgery, and Meniere’s disease (secondary BPPV) (7, 8). However, BPPV is predominantly idiopathic (90%) (3, 9). Despite the terminology being used with BPPV, this condition might not be so benign after all, as it may have a severe, negative impact on the quality of life (10, 11) with adverse psychosocial consequences such as avoidance behavior (interruption of car driving (24%) and social isolation (18%) (3)), sick leave (24%) (3), and depression (78% in the elderly) (12).

The precise pathophysiology of BPPV remains unclear. The leading theory describes BPPV as a mechanical disorder of the vestibular organ that is caused by displacement of otoconia from the macula of the utricle into one or several of the semicircular canals (SCCs) (8). Unfortunately, to the best of our knowledge, only a sparse number of publications provide hard proof of this displacement of otoconia from the utricle into the SCCs (13–17). However, the current prevailing models for the impact of displaced otoconia on canal function, canalolithiasis, and cupulolithiasis agree with many patients’ symptoms and nystagmus reported in daily clinical practice. Canalolithiasis describes the condition characterized by free-floating otoconia within the lumen of the SCCs, inducing endolymphatic flow (and associated cupula deflection) when the head moves in such a way that the orientation of the affected SCC changes relative to gravity. The aberrant signal from the cupula creates a perception of angular head movement (vertigo) and positional nystagmus in the plane of the affected SCC (15). Cupulolithiasis describes the condition where otoconia adhere to the cupula, thereby increasing its mass and making it sensitive to changes in orientation relative to gravity. When the orientation of the affected SCC changes relative to gravity by a change in head orientation, the heavy cupula is pulled downward, causing the perception of vertigo and positional nystagmus (lasting until central adaptation causes them to fade) (18). However, the pathophysiology of BPPV, especially the cupulolithiasis model, is still controversial, and there exist several additional theories, such as canalith jam and periampullary canalolithiasis (19–21). BPPV may affect one or several of the SCCs. Location-wise, the posterior SCCs are considered the most common site (48–79%), followed by the lateral SCCs (17–46%) and the anterior (1–3%) SCCs (6, 22–25). Multicanal BPPV (3–20%) describes the condition with concomitant affection of multiple SCCs (ipsilateral and/or bilateral) (6, 25–27).

BPPV diagnostics is based on the inner ear anatomy and the current knowledge and understanding of BPPV pathophysiology. The diagnostic procedures position the patient’s head in such a way that the gravity vector is in the plane of the examined SCC, allowing any displaced otoconia (free-floating or adhered to the cupula) to elicit a positional nystagmus that is typical for the specific type of BPPV. The traditional and most frequently used positional tests include the Dix-Hallpike test (DHT) examining the vertical SCCs (ipsilateral posterior SCC and contralateral anterior SCC) and the Supine Roll test (SRT) (the McClure-Pagnini test) (examining the lateral SCCs) (1, 2, 28). A key diagnostic criterion is that the observed positional nystagmus must agree with the stimulation of the SCC(s) being tested. To increase the diagnostic accuracy, the patient must also experience positional vertigo. However, existing diagnostic criteria do not fully agree on when this positional vertigo must appear. According to the Bárány Society criteria (1) and the Japan Society for Equilibrium Research (28), patients must report positional vertigo in their patient history, but not necessarily during diagnostic testing. In addition to this criterion, the criteria stated by the American Academy of Otolaryngology-Head and Neck Surgery also require positional vertigo associated with the observed nystagmus during the diagnostic testing (2). The diagnostic procedure(s) of BPPV can provide information about (1) laterality (right or left labyrinth), affected SCC(s), and subtype, which is crucial knowledge for choosing the optimal treatment maneuver and, ultimately, BPPV resolution.

Traditional manual diagnostics (TMD) involve low-cost, non-invasive procedures performed manually on-site, utilizing only an examination bed and no specialized equipment. This accessibility makes BPPV diagnostics widely applicable across several healthcare providers, including general practitioners, neurologists, otorhinolaryngologists (ENTs), geriatricians, physiotherapists, and emergency departments. Despite these advantages, performing the BPPV diagnostics properly can prove challenging. The core of this challenge might be a combination of: (1) the anatomical variance of the SCC orientation (29, 30), (2) the inter- and intra-examiner variability in obtaining the precise head orientation and - movement during the TMD (31, 32), (3) the inter-individual variation of the level of cooperation and/or physical limitations (e.g., high BMI, impaired neck mobility) (33, 34), (4) the heterogeneous nature of BPPV variability of the exact position(s) of the otoconia, their size and quantity within the SCC(s) (16, 35–37), and (5) the level of experience with identification and interpretation of positional nystagmus (noting that up to 71–88% of individuals without BPPV exhibit some form of positional nystagmus during BPPV diagnostics) (38, 39). To some extent, technological advancements, such as mechanical rotation chairs (MRCs), have overcome some of these challenges with BPPV diagnostics. MRCs enable standardized, controlled multi-planar (pitch-, roll-, and yaw axes) 360° head (on-body) rotations that allow precise head orientation related to the (assumed) orientation of the SCCs. In addition to enhancing the reproducibility and accuracy of the diagnostic tests, diagnostics with an MRC reduce the diagnostic inaccuracy induced by the lack of cooperation and the general condition of the patient. Compared to TMD, MRC diagnostics seem to be more sensitive to the detection of BPPV, particularly in patients with reduced cooperation or with non-posterior canal BPPV (33, 34). Furthermore, it was shown that the amplitude of the head orientation in the manual BPPV treatment maneuvers (Epley) imposed by experienced clinicians is inaccurate (inaccuracies of ±20–30 degrees were reported) (40). This supports the hypothesis that the precise head orientation established in the MRC, compared to inaccuracies in the imposed head orientation by TMD, might explain the higher sensitivity of the MRC diagnostics compared to the TMD. However, the influence of the head orientation and -movements in manual BPPV diagnostics has, to our knowledge, not yet been investigated.

This study aims to address this knowledge gap using a randomized controlled crossover design. The primary objective was to examine the association between the outcome of TMD and the head orientation and -movements. The results from the MRC diagnostics (performed by the same examiner) serve as the gold standard. Additionally, this study aims to explore whether a critical window (where the chance of a correct diagnostic outcome is optimal) can be determined for (1) head orientation and (2) head movements (angular velocity and duration of movement) in the TMD.

## 2 Materials and methods

### 2.1 Study design

This study employed a randomized, controlled, open-label crossover design, adhering to the Consolidated Standards of Reporting Trials (CONSORT) statement for crossover trials (41) and incorporating relevant elements from the Standards for the Reporting of Diagnostic Accuracy Studies (STARD) (42). This research formed a part of a broader umbrella study overall investigating BPPV diagnostics and, therefore, sharing participant data with two previously published studies: one comparing diagnostic modalities (TMD and MRC diagnostics) (34) and another quantifying the head orientation and - movement during TMDs (31). Due to the exploratory nature of this specific study and the absence of comparable prior research, an independent power calculation was not performed. Instead, the sample size was determined based on the *a priori* power calculation of the comparative diagnostic study (34).

All participants underwent BPPV diagnostics using both TMD and MRC diagnostics, with the order of the diagnostic methods randomized in a 1:1 ratio. The randomization was achieved using permuted blocks 4, 6, and 8 (made with Sealed Envelope Ltd. 2022). To minimize potential carryover effects, such as fatigue of the positional nystagmus and vertigo, participants were seated for a minimum of 30 min between the two diagnostic methods (43). While blinding of the examiner and participants was not feasible due to the nature of the interventions, the examiner was kept blinded [no feedback of the output of the head-mounted sensors (inertial measurement unit), see 2.3.2 materials] to the specific head orientation that were imposed in the TMDs.

### 2.2 Participants and setting

The study was conducted between April 12, 2023, and January 11, 2024, at a university hospital-based tertiary outpatient clinic specializing in vestibular Disorders (the Balance and Dizziness Centre, Department of Otorhinolaryngology, Head and Neck Surgery and Audiology, Aalborg University Hospital, Denmark). Participants were referred by general practitioners within the North Denmark Region and private ENT practices in the North and Central Denmark Regions. The general practitioners were instructed to refer patients with a typical BPPV case history without performing any canalith repositioning maneuvers before referral. In contrast, participants referred by the private ENT practices had undergone one or several unsuccessful canalith repositioning maneuvers at the time of referral.

The same examiner screened all referred patients for eligibility at their initial visit. Inclusion criteria included an age of 18 years or above, a typical BPPV case history, including short-lived positional vertigo (typically lasting less than 1 min, with a maximum duration of a few minutes), and sufficient Danish proficiency (both written and spoken) to understand the informed consent. Exclusion criteria included spontaneous- and/or gaze-evoked nystagmus, ejection fraction < 40%, known cerebral aneurysm, recent cerebrovascular event (< 3 months), arterial dissection disease, pregnancy, neck and/or spine immobility impeding the TMDs, physical limitations excluding MRC diagnostics (weight ≥ 150 kilograms and/or height ≥ 2 meters), insufficient cooperation during the diagnostic testing (TMDs and/or MRC diagnostics), and intake of sedative antihistamines within the past 7 days. Eligible participants received oral and written information and provided written consent before enrollment.

All diagnostic tests were performed by the same (right-handed) examiner, who had prior experience with conducting and interpreting TMD tests equivalent to that of a basic junior ENT resident. Prior to participant enrollment, the examiner received additional BPPV management training, which was in accordance with the level of training for the health professionals managing BPPV at the study site (a tertiary center for Dizziness and Balance). This training encompassed the use of the equipment (including operation of the MRC), identification and interpretation of nystagmus, and training in doing TMDs. Supervision was provided by two neurotology experts throughout the study period (DDH and HK), with scheduled sessions at the beginning and end of the study period. *Ad hoc* supervision was provided upon request by the examiner.

### 2.3 Materials

#### 2.3.1 Videonystagmography

Eye movements during the TMDs and MRC diagnostics were visualized and recorded using videonystagmography (VNG) goggles (VF405®, Interacoustics©, Middelfart, Denmark) with infrared light. To eliminate the participants’ visual reference, the VNG goggles were covered during the diagnostic testing. The VNG goggles, connected to the accompanying software (Micromedical VisualEyes™, version 3.1.0.203, Interacoustics©, Middelfart, Denmark), enabled characterization of nystagmus (vertical, horizontal, and/or torsional) and quantification of the average slow-phase velocities (of vertical and horizontal nystagmus). The eye images (in black and white) were displayed on a 55-inch wall-mounted screen.

#### 2.3.2 Inertial measurement unit

**Figure 1:**
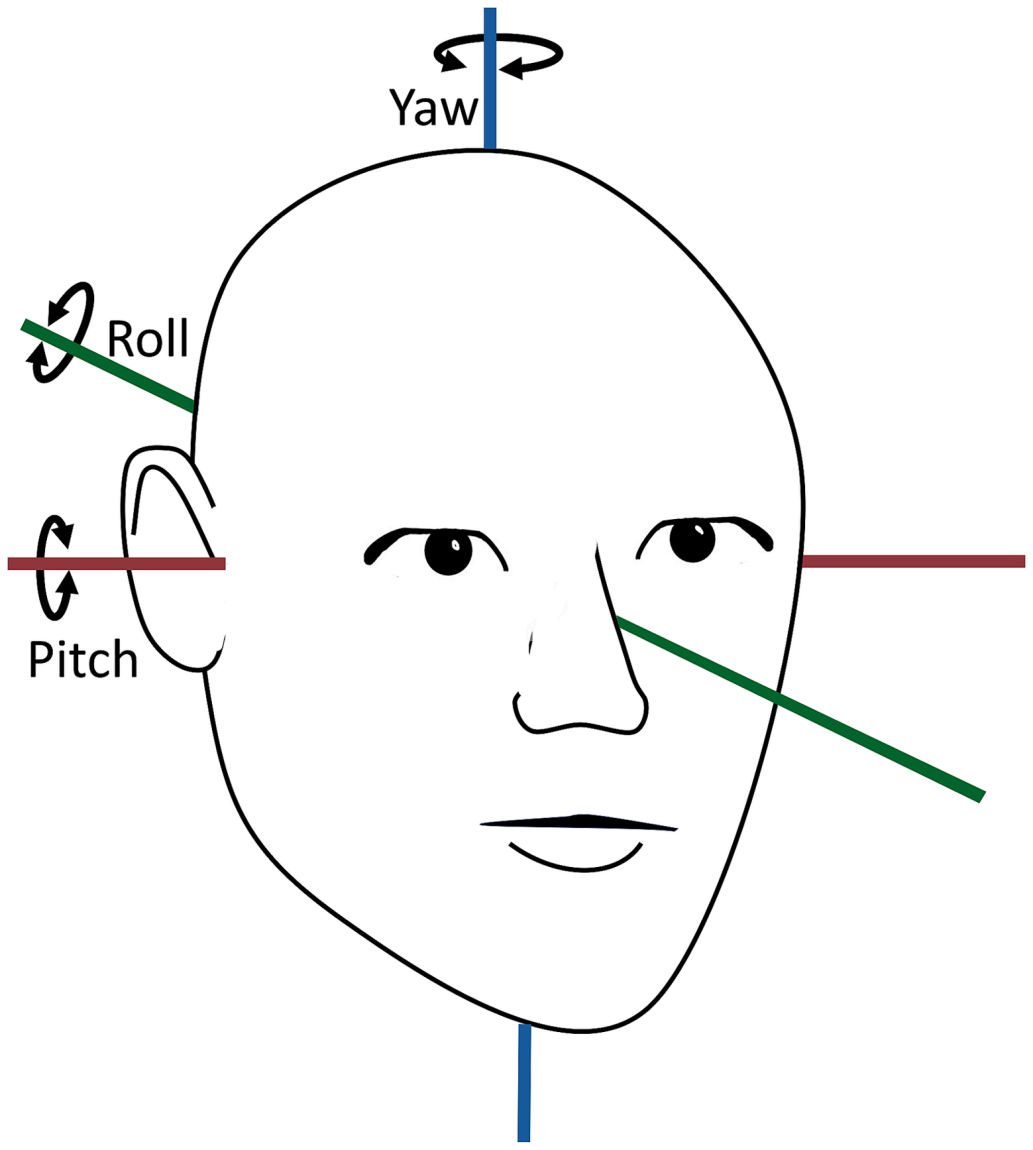
Overview of yaw-, pitch-, and roll axes of the human head orientation. The head movement around the yaw axis is horizontal, the head movement around the pitch axis is vertical, and the head movement around the roll axis is torsional. The figure is modified according to (31).

The VNG goggles were fitted with a six-degree-of-freedom inertial measurement unit (6DOF IMU) (VORTEQ™, Interacoustics©, Middelfart, Denmark) This 6DOF IMU, integrating a 3-axes accelerometer and a 3-axes gyroscope, recorded the head orientation and -movements in real-time. The data transmission to the software was wireless via Bluetooth (250 Hz sampling rate), with a cable connection serving as a backup in the event of technical issues (500 Hz sampling rate). The 6DOF IMU output consisted of the rotation of the head in quaternions (one scalar component (w) and three vector components (x, y, and z)), representing the rotation of the head around the pitch- (x), roll- (y), and yaw (z) axes (Figure 1). To analyze the 6DOF IMU data, the quaternions were converted to Euler angles (44). The 6DOF IMU sensor was meticulously tested prior to data collection to ensure that the data output agreed with the actual orientation and movements performed. The testing included 90° and 360° rotations around the pitch-, roll-, and yaw axes (referenced as when fixated on top of the VNG goggles). Due to frequent slippage of the VNG goggles during the MRC diagnostics, the 6DOF IMU data with MRC diagnostics was not collected.

#### 2.3.3 Software to process and display the inertial measurement unit data

Custom software, developed in collaboration with biomedical engineering students, processed and displayed the 6DOF IMU data collected during the TMDs. The software differentiated between the stationary head orientation (static phases) and the head movement (dynamic phases) during the SRT and the DHT. A dynamic phase was defined as head movements exceeding a 13° threshold within 120 samples in a pitch- or yaw-axis rotation. In the static phases, the software calculated the mean head angles (°). For the dynamic phases, the software calculated the mean and peak angular velocity (°/second) along with the total duration of the head rotation (seconds). The data was visualized as graphs of the head orientation over time. The software displayed the supine position alongside the right and left SRTs in a single window and the DHT data in separate windows for each side. Manual calculation of the variables was performed when the software was unable to distinguish between the static- and dynamic phases.

#### 2.3.4 Mechanical rotation chair

The MRC diagnostics were performed using the Thomas Richard-Vitton Repositional Chair (TRV Chair®, Interacoustics©, Middelfart, Denmark), a biaxial MRC with two 360° rotational frames with lockable preset positions, enabling a total 360° vertical and horizontal rotation, which, depending on the starting position, allows rotations around all three axes (pitch-, roll-, and yaw axes). The examiner operated the MRC manually while the participants were secured with a four-point harness, head fixation, and foot support.

### 2.4 Intervention

Prior to the BPPV diagnostics, participants were screened for spontaneous- and gaze-evoked nystagmus and for the presence of a vestibulo-ocular reflex, including a fixation-suppression test (manual to the left and right yaw-axis rotation in the MRC with and without visual fixation). Participants with abnormal results were excluded and referred for further evaluation following local clinical guidelines. Neither additional vestibular (no video head impulse test was performed to avoid potential displacements of otoliths) nor neurological examination was performed. The TMD and MRC diagnostics were conducted in the same standardized sequence of positions: (1) upright position with the head in neutral position, (2) supine position (30 s), (3) right SRT, followed by (4) left SRT (right and left SRT were held until nystagmus was observed and interpreted, or for a maximum of 30 s if no nystagmus appeared), (5) upright position with the head in neutral position, (6) upright position with the head rotated 45° to the right, (7) right DHT position (60 s), (8) upright position with the head in neutral position, (9) upright position with the head rotated 45° to the left, (10) left DHT position (60 s), and (11) upright position with the head in neutral position. Based on recommendations from simulation tests, the sequence of diagnostic tests was chosen to minimize the risk of displacement of otoconia debris in the lateral SCC during the DHT (45).

**Figure 2:**
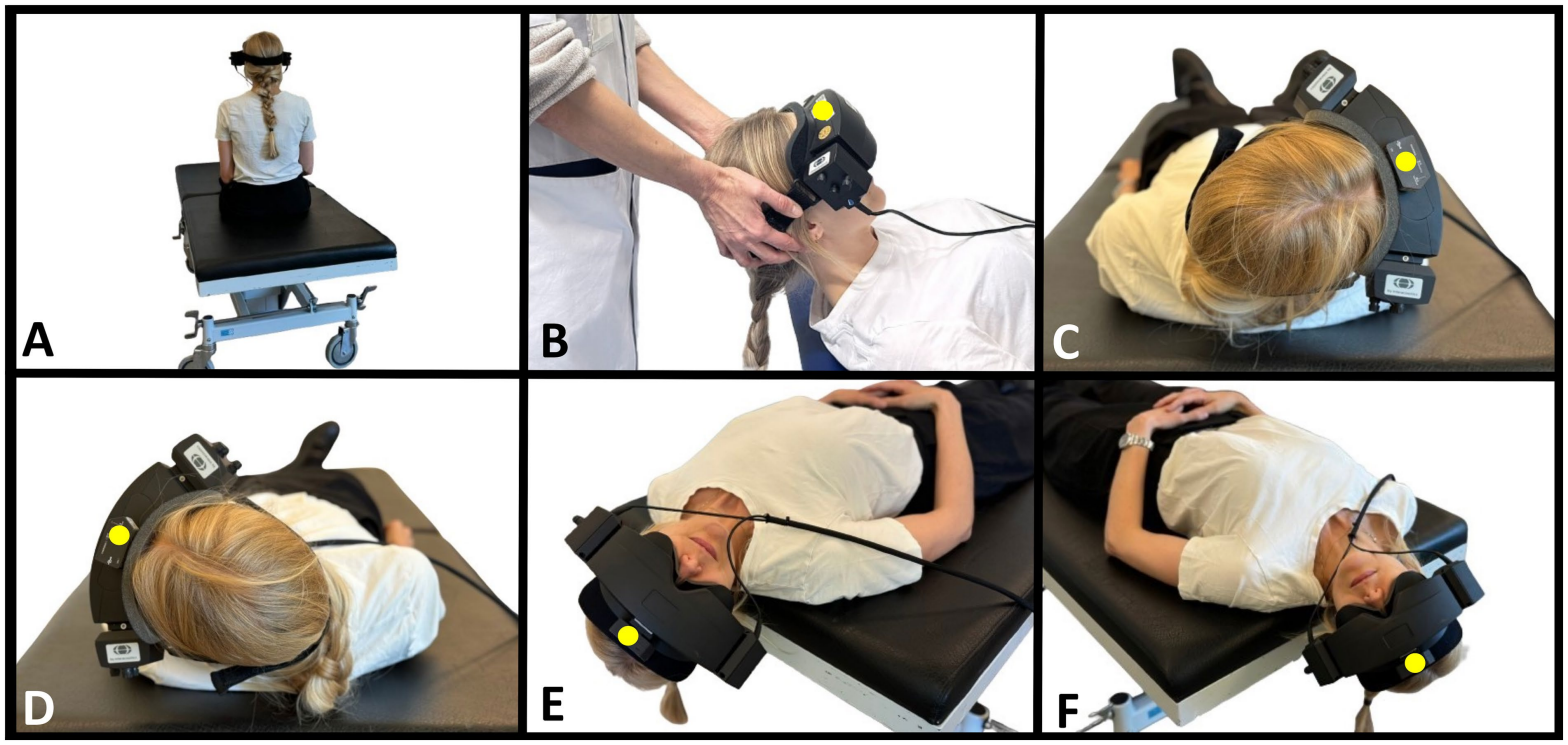
Traditional manual BPPV diagnostics. **(A)** Starting position with the participant sitting upright on the examination bed. Head is in neutral position (guided by the examiner) and fitted with videonystagmography goggles. **(B)** Supine position with the neck flexed 30° (corresponding to a − 60° rotation around the pitch axis from the starting position). Examiner in cranial position. **(C)** Right Supine Roll test. From supine position, the participant’s head is rotated 90° to the right (90° yaw-axis head rotation). **(D)** Left Supine Roll test. From the right Supine Roll test, the head is rotated 180° to the left (end position corresponds to −90° yaw-axis head rotation from the starting position). **(E)** Right Dix-Hallpike test. From the upright sitting position (A), the head is rotated 45° to the right (45° yaw-axis head rotation), followed by a backward movement to supine position with the neck extended 30° below the horizontal plane (−120° pitch-axis head rotation). **(F)** Left Dix-Hallpike test. From the upright sitting position (A), the head is rotated 45° to the left (−45° yaw-axis head rotation), followed by a backward movement to supine position with the neck extended 30° below the horizontal plane (−120° pitch-axis head rotation). A six-degree-of-freedom inertial measurement unit is attached to the top of the videonystagmography goggles (marked with a yellow dot). The figure is modified according to (31, 34).

**Figure 3:**
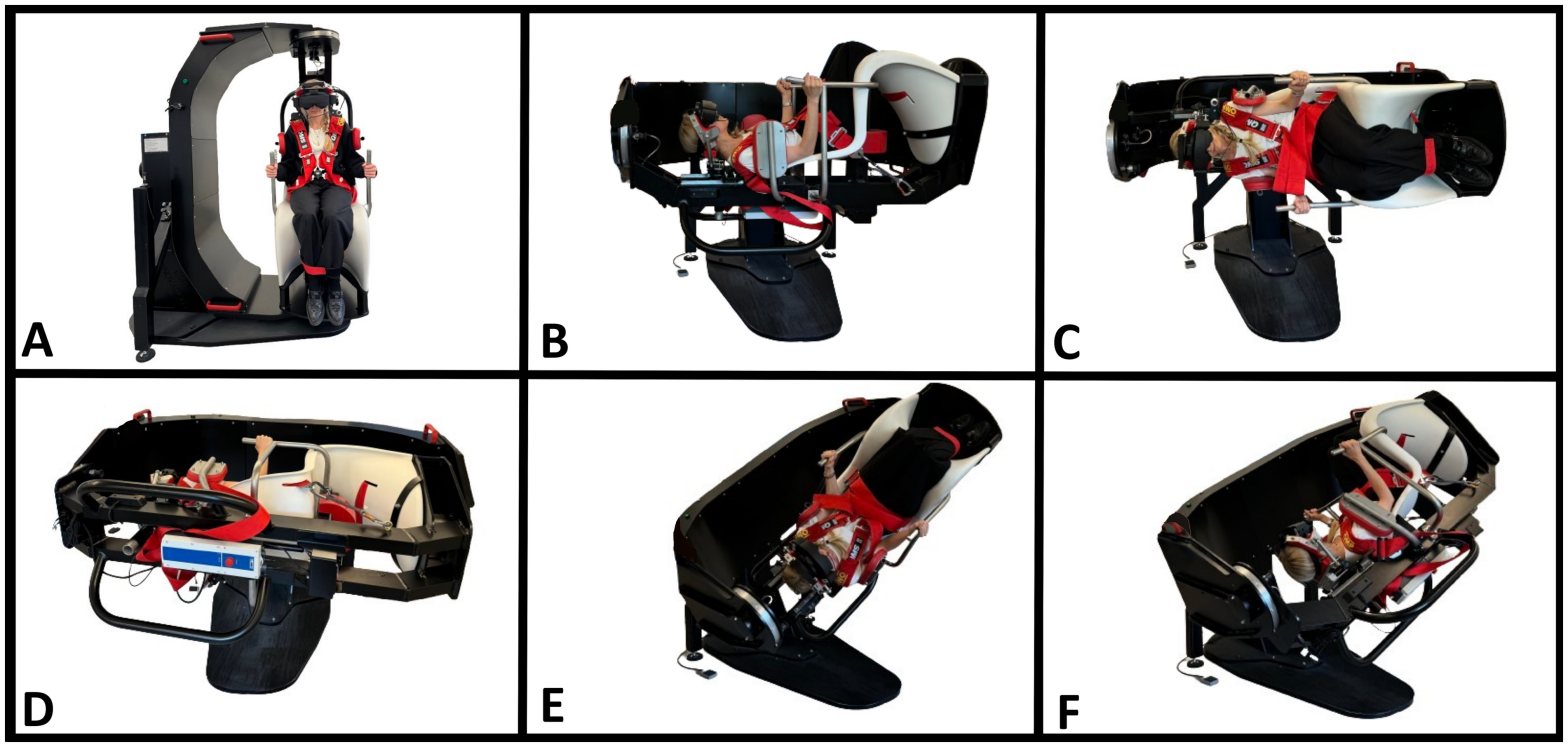
BPPV diagnostics with a mechanical rotation chair (MRC). **(A)** Starting position. The participant is seated in an upright position and fitted with videonystagmography goggles. **(B)** Supine position. The MRC is rotated 90° backward in the pitch plane of the MRC with the participant’s neck flexed approximately 30° with an integrated headrest (−60° pitch-axis head rotation). **(C)** Right Supine Roll test. The MRC is rotated 90° to the right in the MRC’s roll plane (90° yaw axis head rotation). **(D)** Left Supine Roll test. The MRC is rotated 180° to the left in the MRC’s roll plane (−90° yaw-axis head rotation). **(E)** Right Dix-Hallpike test. From the starting position **(A)**, the MRC is rotated 45° to the right in the MRC’s yaw plane, followed by a 120° backward rotation in the MRC’s pitch plane (45° yaw-axis and −120° pitch-axis head rotation). **(F)** Left Dix-Hallpike test. From the starting position **(A)**, the MRC is rotated 45° to the left in the MRC’s yaw plane, followed by a 120° backward rotation in the MRC’s pitch plane (−45° yaw-axis and −120° pitch-axis head rotation). The figure is modified according to (34).

The target head angles were based on the Bárány Society diagnostic criteria for BPPV (1) and were consistent for both TMDs (Figure 2) and MRC diagnostics (Figure 3). The SRT preceded the DHT to minimize the potential unintended displacement of otoconia in the lateral SCC during DHT (46). The intended duration of the movement to the primary diagnostic positions (positions 2, 3, 4, 7, and 10) was set to be within 2 seconds. With the MRC diagnostics, the transition between positions 3 and 4 was ideally performed as one single smooth movement. However, in obese participants, this movement was divided into two steps for safety. During diagnostic procedures with the MRC, target positions were ensured by using fixed position markers of the MRC. No visual feedback was provided during the TMDs, thereby allowing only the examiner’s subjective bedside assessment of the participant’s head orientation. To optimize participant cooperation, all participants received detailed pre-test instructions, including the importance of maintaining a straightforward gaze eye position and minimizing blinking (incorporating required reminders). To prevent any eye movement artifacts, the participants were instructed to close their eyes during the head movement preceded by a three-second countdown. For the TMDs, participants were instructed to sit upright on the examination bed (with the legs straight if possible) and with a neutral head orientation (guided by the examiner). Participants were informed that the examiner should perform and determine all head orientation and -movements.

The 6DOF IMU sensor was calibrated at positions 1, 5, and 8 (upright position with neutral head position). Throughout the TMDs, the examiner maintained a firm grip on the VNG goggle headband to prevent it from displacing. The examiner reviewed eye movements in real-time during all diagnostic tests. The recorded eye videos could be reviewed subsequently (with a supervising neurotologist if needed) to support the diagnostic conclusion. All recorded eye movement files were reviewed post-data collection by a blinded neurotology expert to ensure high standards of diagnostic accuracy (34).

The examiner placed the BPPV diagnosis and further specified (1) laterality, (2) affected SCC(s), and subtype (canalolithiasis or cupulolithiasis) on-site in accordance with the Bárány Society diagnostic criteria (1). A BPPV-characteristic positional nystagmus (BPPV-CPN) was defined as a positional nystagmus with characteristics compatible with having BPPV (specified in Table 1), an average slow-phase velocity of a minimum of 3 °/second, and at least five consecutive beats (47).

**Table 1:** Characteristics of BPPV-CPN.

	BPPV subtype		Positive BPPV diagnostics
	Canalolithiasis	Cupulolithiasis	
	Supine Roll test		
Lateral SCC	Geotropic nystagmus lasting <1 min. No or brief latency.	Apogeotropic nystagmus lasting >1 min. No or brief latency.	Positive bilateral geotropic or apogeotropic BPPV-CPN and typical BPPV case history.
	Ipsilateral Dix-Hallpike test		
Posterior SCC	Upbeating vertical nystagmus with a torsional component (beating toward the lower ear) lasting <1 min. Latency of a few seconds.	Upbeating vertical nystagmus with torsional component (beating toward the lower ear) lasting >1 min. No or brief latency.	Positive BPPV-CPN and positive BPPV case history.
	Contralateral Dix-Hallpike test*		
Anterior SCC	Downbeating nystagmus lasting <1 min. With or without a torsional component (beating toward the lower ear). No or brief latency.	Downbeating nystagmus lasting >1 min. With or without a torsional component (beating toward the lower ear). No or brief latency.	Positive BPPV-CPN and positive BPPV case history.

BPPV-CPN: BPPV-characteristic positional nystagmus; SCC: semicircular canal; Geotropic nystagmus: beating towards the ground; Apogeotropic nystagmus: beating away from the ground. The definition of BPPV-CPN is based upon the nystagmus descriptions stated in the Bárány Society diagnostic criteria (1). The table is modified according to (34). *Involvement of the anterior SCC may elicit a positive BPPV-CPN bilaterally.

The diagnostic outcome was defined as the observation of BPPV-CPN in each side of the SRT and DHTs rather than the BPPV diagnosis itself. The reason for choosing this approach was that previous results from simulation models have shown that the nystagmus patterns with lateral canal BPPV are highly dependent on both the position of the otoconia (the ampullary or non-ampullary arm of the lateral SCC) as well as the initial side of the SRT performed (46). If the BPPV diagnosis (bilateral geotropic or apogeotropic nystagmus) was exclusively used as the study’s outcome measure for lateral canal BPPV, we could theoretically exclude those cases with lateral canal BPPV, where only unilateral BPPV-CPN was observed during the SRT, e.g., due to accidental liberation. However, when displaying the combined result of the positional nystagmus on both the right and left SRT, the term ‘BPPV diagnosis’ will be used.

The BPPV was classified as either primary or secondary BPPV. Secondary BPPV was defined when the participant had a previous or present ipsilateral inner ear disease (excluding presbycusis), previous ipsilateral middle- or inner ear surgery, or recent head trauma (< 6 months prior to the debut of symptoms). Primary BPPV (idiopathic) was defined when no clear etiology was identified. Participants diagnosed with BPPV were offered subsequent treatment with MRC following local clinical guidelines.

### 2.5 Data collection

Data collection was conducted during the participants’ first visit, where the examiner gathered information through electronic patient record review, history taking, and physical examination(s). This information was entered into a secure REDCap® database (version 13.1.37) hosted by the North Denmark Region (48, 49). The 6DOF IMU data were recorded in real-time during the TMDs and saved immediately after the participants’ visit. The processed head orientation data from the 6DOF IMU software was exported as a compound CSV file and subsequently merged with the main REDCap export file within the statistical software (StataNow/MP 18.5), which was used for all data analyses.

### 2.6 Statistical analysis

The baseline characteristics of the randomized groups were summarized using descriptive statistics. Continuous variables were presented with means and standard deviations, with normality assessed visually (using histograms and Q-Q plots) and using the Shapiro–Wilk test. Categorical data were reported using absolute and relative frequencies. The head orientation was displayed using the imposed head angles, and the head movement variables (angular velocity and duration of movement) were displayed as absolute numbers. For clarity, only the relevant head orientations of the SRT and DHT were presented in the tables and figures. The BPPV-CPN observed during the MRC diagnostics was used as the gold standard, based on prior studies showing that the MRC diagnostics appear to be more sensitive than TMDs in detecting BPPV (33, 34). The association between the BPPV-CPN and the head orientation, as well as the head movement variables, was tested by comparing the true positives (cases where the BPPV-CPN observed during the MRC diagnostics was reproduced during the TMD) and false negatives (cases where the BPPV-CPN observed during the MRC diagnostics was not reproduced during the TMD).

Comparison between all groups was performed using an unpaired Student’s t-test (with Welch’s t-test for unequal variances) for continuous data and a Chi-square test (with Fisher’s exact test used for expected cell counts less than 5) for categorical data. In the case of non-normal distribution, bootstrapping was used to obtain reported results for continuous variables. The head orientation during the TMDs was visualized with scatterplots and a head orientation-time graph. The head movement variables during the TMDs were visualized with scatterplots.

All data analyses included only participants who completed the trial and for whom the 6DOF IMU data were of sufficient quality (per protocol analyses). The analysis was not performed blinded to the randomization, and no interim analyses were conducted during the study period. The data analyses and visualization were selected in collaboration with an independently certified biostatistician who also provided consultation throughout the study period. An alpha significance level of 0.05 was used for all statistical tests. All data analyses were performed using StataNow/MP 18.5.

## 3 Results

**Figure 4:**
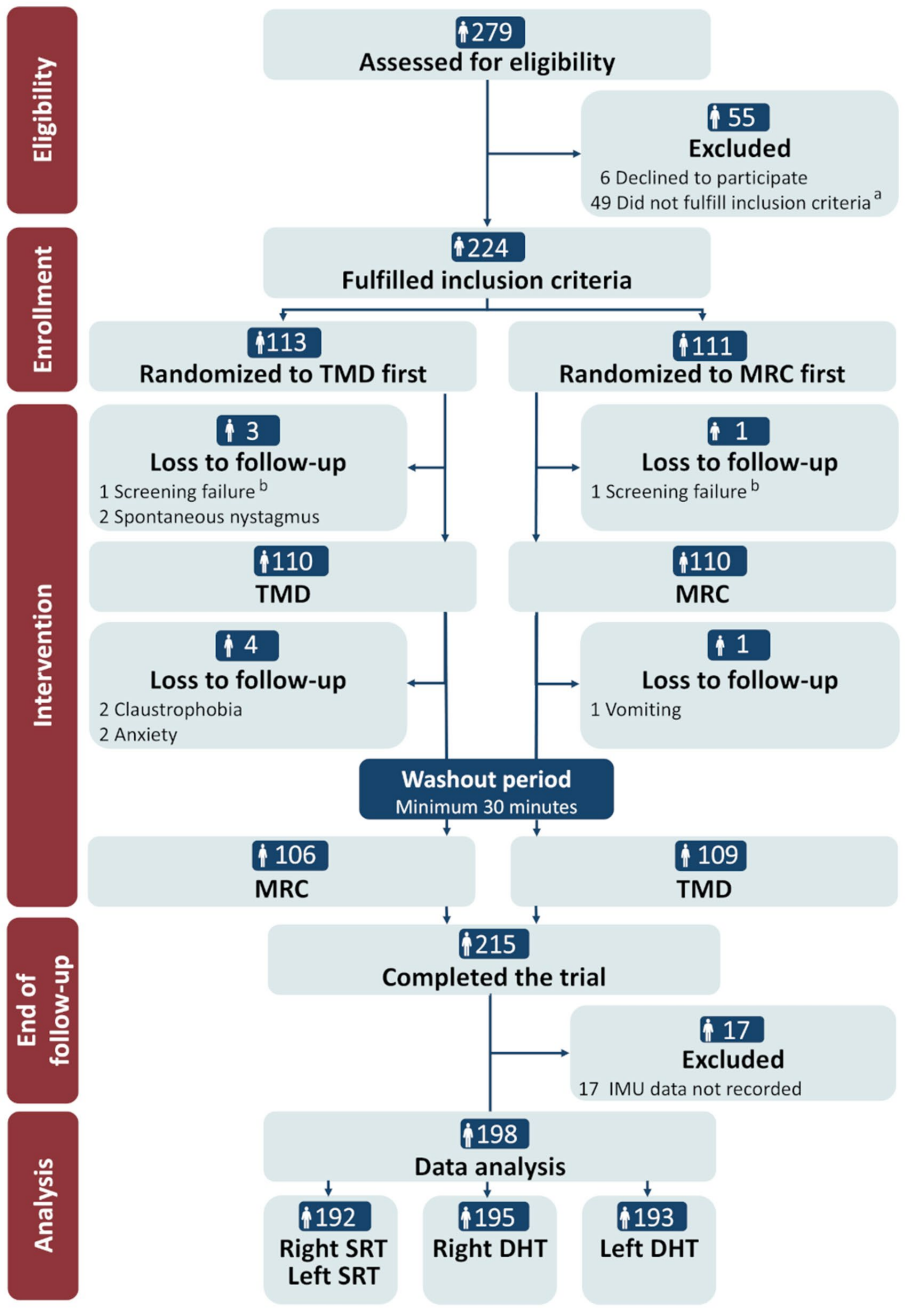
CONSORT flow diagram. TMD, traditional manual diagnostics; MRC, mechanical rotation chair; SRT, Supine Roll test; DHT, Dix-Hallpike test. ^a^ Reasons for not fulfilling the inclusion criteria: remission of vertigo (*n* = 34), neck- and back immobility (*n* = 5), insufficient understanding of Danish language (*n* = 2), spontaneous- and gaze-evoked nystagmus (*n* = 1), intake of sedative antihistamines (*n* = 2), pregnancy (*n* = 1), and cardiovascular comorbidity (*n* = 4). ^b^ Reasons for screening failure: intake of sedative antihistamines (*n* = 1) and cerebral hemorrhage (<3 months) (*n* = 1).

Of the 279 participants assessed for eligibility, 224 (80.3%) met the inclusion criteria and were randomized to the order of the TMD and MRC diagnostics. Spontaneous remission of the vertiginous symptoms (*n* = 35, 61.8%) was the main reason for exclusion (see Figure 4 for other reasons). Nine participants (4.0%) were lost to follow-up due to screening failure (*n* = 2), spontaneous nystagmus (*n* = 2), or reasons related to the MRC diagnostics (vomiting: *n* = 1; anxiety: *n* = 2; claustrophobia: *n* = 2), resulting in a 96.0% completion rate (*n* = 215). Data from 17 participants were excluded prior to data analysis due to universal insufficiency of 6DOF IMU data, leaving 198 participants for the analysis. A further minor reduction occurred for the right and left SRT (*n* = 192) and the right and left DHT (*n* = 195/193, respectively) in the data analysis due to insufficient 6DOF IMU data quality for the specific tests (Figure 4).

Baseline characteristics are shown in Table 2. The 198 participants had a mean age of 58.4 years (range: 18–91) and a female-to-male ratio slightly above 2:1 (female: 70.7%). Overall, BPPV was confirmed in 54.5% (*n* = 108) of the participants. The BPPV confirmation rate was considerably higher (74.1%) among the 27 participants (13.6%) referred by private ENT clinics compared to the 171 participants (86.4%) referred by general practitioners (BPPV confirmation: 51.5%).

**Table 2:** Baseline characteristics (*n* = 198).

	Total (*n* = 198)		Groups of randomization				*p*-value
			Randomized to MRC diagnostic first (*n* = 98)		Randomized to TMD first (*n* = 100)		
Age, mean (sd)	58.4	(16.0)	57.5	(14.4)	59.4	(17.4)	0.410
Female, *n* (%)	140	(70.7)	69	(70.4)	71	(71.0)	0.927
Referred from:							
General practitioner, *n* (%)	171	(86.4)	88	(89.8)	83	(83.0)	0.164
Private ENT, *n* (%)	27	(13.6)	10	(10.2)	17	(17.0)	-
BPPV diagnosis total*, yes, *n* (%)	108	(54.6)	48	(49.0)	60	(60.0)	0.119
BPPV diagnosis with TMD, yes, *n* (%)	79	(39.9)	38	(38.8)	41	(41.0)	0.749
BPPV diagnosis with MRC, yes, *n* (%)	100	(50.5)	44	(44.9)	56	(56.0)	0.118
Etiology of BPPV:	n = 108		n = 48		n = 60		
Idiopathic BPPV, *n* (%)	90	(83.3)	40	(83.3)	50	(83.3)	1.000
Secondary BPPV, *n* (%) ^a^	18	(16.7)	8	(16.7)	10	(16.7)	-
BPPV characteristics ^b^	n = 100		n = 44		n = 56		
Monocanal BPPV, *n* (%)	80	(80.0)	33	(75.0)	47	(83.9)	0.268
Posterior canal BPPV, *n* (%)	64	(64.0)	25	(56.8)	39	(69.6)	0.185
Lateral canal BPPV, *n* (%)	15	(15.0)	7	(15.9)	8	(14.3)	0.821
Anterior canal BPPV, *n* (%)	1	(1.0)	1	(2.3)	0	(0.0)	-
Multicanal BPPV, *n* (%)	20	(20.0)	11	(25.0)	9	(16.1)	0.268
Bilateral posterior canal BPPV, *n* (%)	10	(10.0)	5	(11.4)	5	(8.9)	0.745
Unilateral posterior and lateral canal BPPV, *n* (%)	10	(10.0)	6	(13.6)	4	(7.1)	0.328

MRC, Mechanical rotation chair; TMD, Traditional manual diagnostics; ENT: Otorhinolaryngologist. *BPPV diagnosed total’ refers to the number of participants with BPPV diagnosed with TMD and/or MRC diagnostics. ^a^Secondary BPPV: head trauma (*n* = 13), ipsilateral previous vestibular neuritis (*n* = 18), ipsilateral previous middle- or inner ear surgery (*n* = 5). ^b^BPPV characteristics are based upon the MRC diagnostics (considered the gold standard with this study). All p-values are obtained by unpaired t-test (Welch’s test: unequal variance) and chi2-test (Fisher’s exact: expected cell count <5). A *p*-value < 0.05 is considered significant (*). Please note that there are no significant differences between the two randomized groups.

Of the total number of participants diagnosed with BPPV (*n* = 108), diagnostics with an MRC detected a higher number of BPPV (*n* = 100, 92.6%) than TMDs (*n* = 79, 73.1%). Posterior canal BPPV was the most frequent location (64.0%), followed by lateral canal BPPV (15.0%) and anterior canal BPPV (1.0%). Multicanal BPPV (20.0%) comprised equal proportions of bilateral posterior canal BPPV (10.0%) and ipsilateral posterior and lateral canal BPPV (10.0%). All baseline characteristics were similar across the randomized groups.

### 3.1 Head orientation in the manual SRT and DHT

With TMDs, all head angles deviated significantly from the target head angles (the confidence interval did not include the target head angle) (Table 3). This inaccuracy was greatest for the yaw-axis head angles of the SRTs, which were considerably undershot. During TMDs, BPPV-CPN was observed in 8.9% (*n* = 17/192) of the right SRTs, 10.9% (*n* = 21/192) of the left SRTs, 24.1% (*n* = 47/195) of the right DHTs, and 15.5% (*n* = 30/193) of the left DHTs. The left SRT yaw-axis head angle was significantly more accurate in the group with observed BPPV-CPN than in the group without BPPV-CPN. No other significant differences in SRT and DHT yaw- and pitch axes were found between groups with and without observed BPPV-CPN (Table 3).

**Table 3:** Head orientation and BPPV-characteristic positional nystagmus with traditional manual diagnostics (*n* = 198).

	Target head angle	Imposed head orientation with TMD						*p*-value
		Total		BPPV-CPN		No BPPV-CPN		
		Mean	(95% CI)	Mean	(95% CI)	Mean	(95% CI)	
Supine roll test								
*Right side*		*n* = 192		*n* = 17		*n* = 175		
Yaw axis, °	90.0	70.3	(68.7, 71.9)	69.7	(64.4, 75.0)	70.4	(68.7, 72.0)	0.791
Pitch axis, °	−60.0	−64.4	(−65.8, −63.1)	−67.2	(−71.1, −62.8)	−64.2	(−65.5, −62.8)	0.156
*Left side*		*n* = 192		*n* = 21		*n* = 171		
Yaw axis, °	−90.0	−66.2	(−67.7, −64.6)	−71.2	(−77.0, −65.3)	−65.6	(−67.1, −64.0)	0.023*
Pitch axis, °	−60.0	−63.4	(−64.7, −62.2)	−64.5	(−68.4, −60.6)	−63.3	(−64.6, −62.0)	0.549
Right Dix-Hallpike test								
*Supine position*		*n* = 195		*n* = 47		*n* = 148		
Yaw axis, °	45.0	47.4	(46.2, 48.7)	46.4	(43.8, 48.9)	47.8	(46.3, 49.3)	0.324
Pitch axis, °	−120.0	−112.2	(−113.8, −110.7)	−111.1	(−114.2, −107.9)	−112.6	(−114.4, −110.8)	0.390
Left Dix-Hallpike test								
*Supine position*		*n* = 193		*n* = 30		*n* = 163		
Yaw axis, °	−45.0	−33.3	(−34.6, −31.9)	−34.6	(−37.9, −31.2)	−33.0	(−34.4, −31.6)	0.417
Pitch axis, °	−120.0	−111.3	(−112.8, −109.8)	−109.9	(−113.5, −106.2)	−111.6	(−113.2, −109.9)	0.417

BPPV-CPN: BPPV-characteristic positional nystagmus; TMD: traditional manual diagnostics. All *p*-values are obtained by unpaired t-test (Welch’s test: unequal variance). A p-value < 0.05 is considered significant (*). Please note that, except for the yaw-axis head angle with the left Supine Roll test, there were no statistically significant intergroup differences in the mean head angles with TMD between groups with and without observed BPPV-CPN.

The TMDs yielded a substantial number of false negatives (cases where the BPPV-CPN observed during the MRC diagnostics was not reproduced during the TMD) (right SRT: *n* = 19; left SRT: *n* = 24; right DHT: *n* = 13; left DHT: *n* = 13) (Table 4). Conversely, a smaller number of participants exhibited BPPV-CPN during the TMD that was not reproduced during the MRC diagnostics (right SRT: *n* = 6; left SRT: *n* = 6; right DHT: *n* = 6; left DHT: *n* = 3).

**Table 4:** Head orientation and true positive or false negative BPPV-characteristic positional nystagmus with traditional manual diagnostics (*n* = 198).

	Target head angle	Imposed head orientation with TMD				p-value
		True positive BPPV-CPN		False negative BPPV-CPN		
		Mean	95% CI	Mean	95% CI	
Supine roll test (*n* = 192)						
*Right side*		*n* = 11		*n* = 19		
Yaw axis, °	90.0	66.4	(59.1, 73.7)	69.3	(63.6, 75.1)	0.512
Pitch axis, °	−60.0	−69.0	(−73.8, −64.2)	−63.5	(−68.8, −58.3)	0.128
*Left side*		*n* = 15		*n* = 24		
Yaw axis, °	−90.0	−69.8	(−77.6, −61.9)	−60.5	(−65.5, −55.5)	0.034*
Pitch axis, °	−60.0	−65.0	(−70.0, −60.0)	−63.5	(−68.1, −58.9)	0.661
Right Dix-Hallpike test (*n* = 195)						
*Supine position*		*n* = 41		*n* = 13		
Yaw axis, °	45.0	47.4	(44.8, 50.1)	45.3	(41.6, 49.0)	0.406
Pitch axis, °	−120.0	−110.8	(−114.3, −107.2)	−111.0	(−118.3, −103.6)	0.958
Left Dix-Hallpike test (*n* = 193)						
*Supine position*		*n* = 27		*n* = 13		
Yaw axis, °	−45.0	−34.8	(−38.5, −31.2)	−33.3	(−41.6, −25.1)	0.748
Pitch axis, °	−120.0	−109.3	(−113.0, −105.6)	−116.2	(−123.5, −108.9)	0.055

BPPV-CPN: BPPV-characteristic positional nystagmus; TMD: traditional manual diagnostics. True and false BPPV-CPN were determined by the BPPV-CPN observed during the diagnostics with a mechanical rotation chair (gold standard). All p-values are obtained by unpaired t-test (Welch’s test: unequal variance). A *p*-value < 0.05 is considered significant (*). Please note that there were no statistically significant intergroup differences in mean head angle between the true positive and false negative BPPV-CPN groups (reference set as BPPV-CPN observed in diagnostics with mechanical rotation chair) except for the yaw-axis head angle with the left Supine Roll test.

Except for the left SRT yaw-axis head angle, no significant difference in the head angles was observed between the true positives and false negatives (Table 4). The left SRT yaw-axis head angle was significantly more accurate (closer to the target head angle: −90°) in the true positives [−69.8° (95% CI: −77.6°, −61.9°)] compared to the false negatives [−60.5° (95% CI: −65.5°, −55.5)].

**Figure 5:**
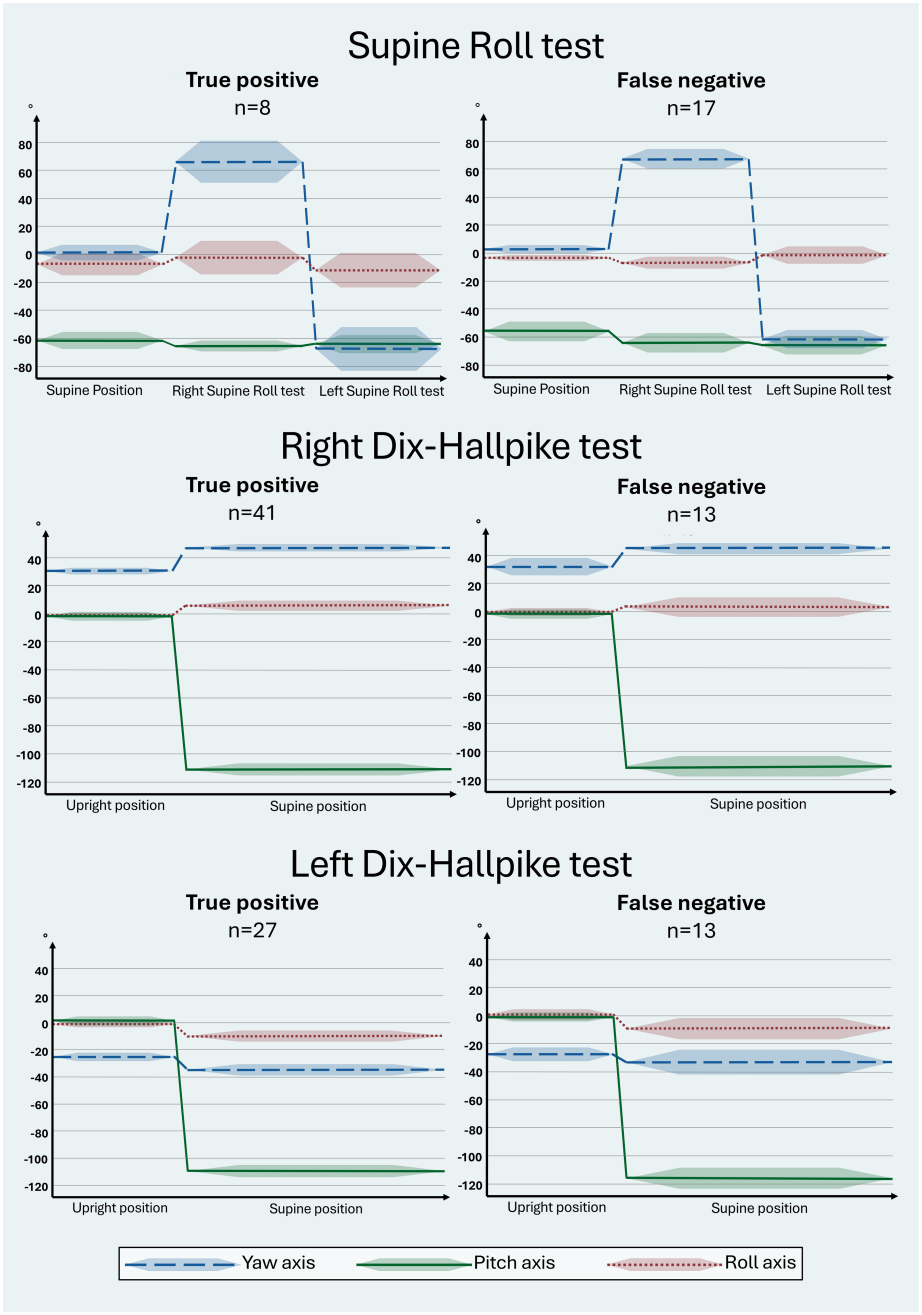
Head orientation with traditional manual diagnostics in relation to the true positive and false negative BPPV diagnosis. The head orientation-time graphs illustrate the yaw- (blue), pitch- (green), and roll (red) axes of the head orientation (mean: solid line; 95% confidence interval: shaded area). Prerequisites for a BPPV diagnosis include bilateral BPPV-characteristic positional nystagmus (BPPV-CPN) (geotropic or apogeotropic nystagmus) with the Supine Roll test (SRT) or torsional upbeating nystagmus with the Dix-Hallpike test (DHT) (Table 1). The reference (true/false) was defined by the outcome of the corresponding test with mechanical rotation chair diagnostics (gold standard). The direction of the head movement around the individual axes is indicated as follows: yaw: + = right, − = left; pitch: + = forward, − = backward; roll: + = right, − = left. For the SRT, the target head orientation was as follows: supine position: yaw: 0°, pitch: −60°, roll: 0°; right SRT: yaw: 90°: pitch: −60°, roll: 0°; left SRT: yaw: −90°, pitch: −60°, roll: 0°. For the DHT, the target head orientation was as follows: upright position: yaw ±45°, pitch 0°, roll 0°; supine position: yaw ±45°, pitch −120°, roll 0°. Please note that there were no statistically significant intergroup differences in the yaw, pitch, and roll axes between groups with true positive and false negative BPPV diagnoses with traditional manual diagnostics for all tests. Please refer to Supplementary Table B for the specific numerical values used in this figure.

The manual SRT and DHT head orientation (including the roll-axis head angle) are visualized in a head orientation-time graph in relation to the BPPV diagnosis, differentiating between the true positives and false negatives in the manual SRT and DHT when compared to the corresponding MRC diagnostic test (Figure 5). Please note that the right and left SRTs are combined (to demonstrate the entire sequence of head orientation during the SRT). Hence, the groups of true positives and false negatives in Figure 5 refer to the BPPV diagnosis combining the observed nystagmus in the right and left SRT (requiring bilateral geotropic or apogeotropic nystagmus for a positive BPPV diagnosis). When using BPPV diagnosis as the outcome for the manual SRT and DHT, there was no significant difference between the yaw, pitch, and roll axes between groups with true positive and false negative BPPV diagnoses. All head orientation-time graphs (Figure 5) revealed a bias in the roll-axis head angle (differing from the target head angle of 0°) for the majority of the SRT and DHT head orientations.

**Figure 6:**
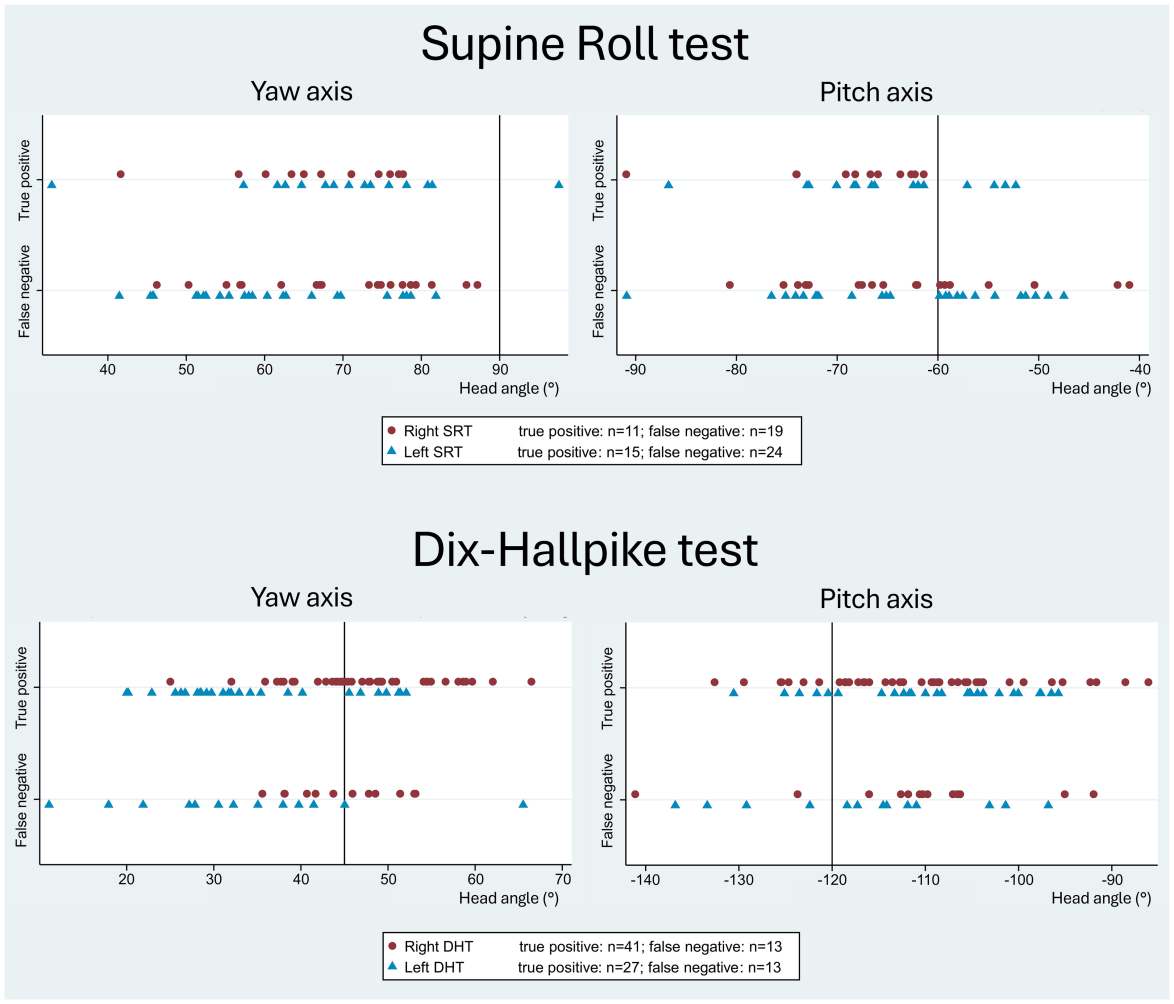
Head orientation with traditional manual diagnostics in relation to the true positive and false negative BPPV-characteristic positional nystagmus. The reference (true/false) was defined by the outcome of the corresponding test with mechanical rotation chair diagnostics (gold standard). The scatterplots display the imposed yaw-axis (left) and pitch-axis head angles (right) with the manual Supine Roll test (SRT) and the manual Dix-Hallpike test (DHT), with symbols indicating test laterality (right side: red circle; left side: blue triangle). With the SRT, the false negative group showed a broader range and greater inaccuracy in both the yaw- and pitch axes compared to the true positive group. With the DHT, the true positive group showed a tendency toward a more pronounced yaw-axis head rotation (right DHT) and pitch-axis head angles clustered between −120° to −100 °, compared to the false negative group.

Figure 6 displays the head orientation in the manual SRT and DHT in relation to the true positive and false negative BPPV-CPN when compared to the corresponding MRC diagnostic tests. The manual SRT head orientation, particularly the left SRT yaw axis, showed an overall broader range and greater inaccuracy of the applied head angles in the false negative group compared to the true positive group. For the SRT pitch axis, the true positives appeared to be clustered between −60° (the target head angle) and −75°. The manual DHT tended to have a more pronounced right DHT yaw-axis head rotation (applied head angle above the target +45°) and pitch-axis head angles clustered between −100° and −120° (right and left DHT) for the true positive BPPV-CPN group compared to the false negative group.

**Figure 7:**
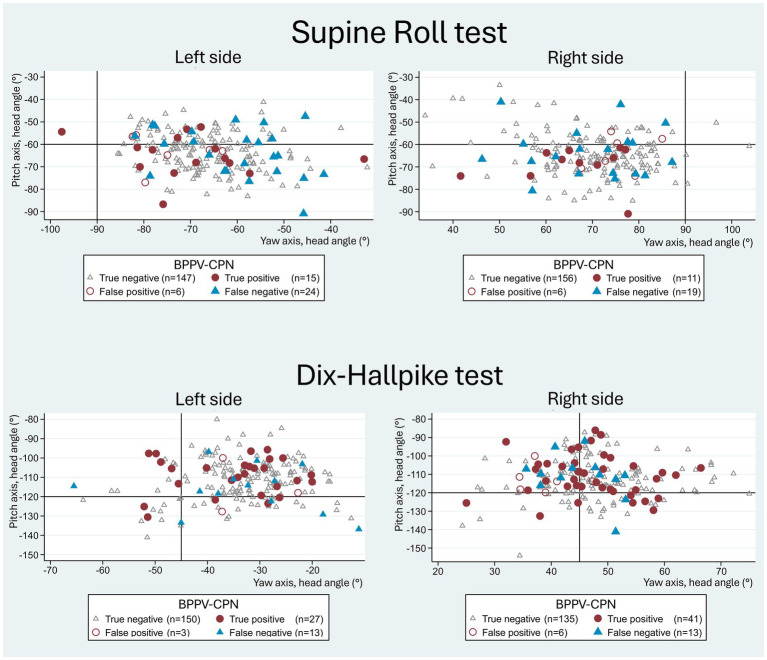
Yaw- vs. pitch-axes with traditional manual diagnostics and BPPV-characteristic positional nystagmus classification. The scatterplots show yaw-axis (x-axis) and pitch-axis (y-axis) head angles with manual Supine Roll test (SRT) and manual Dix-Hallpike test (DHT), with symbols indicating the categories of BPPV-characteristic positional nystagmus (BPPV-CPN): true positive (red circle), false negative (blue triangle), false positive (red hollow circle), and true negative (grey hollow circle). The reference (true/false) was defined by the outcome of the corresponding test with mechanical rotation chair diagnostics (gold standard). The black solid lines represent the target head angles for each axis. Please note that the data points for both the SRT and DHT appear randomly scattered, indicating no clear relationship between the imposed head angles and the BPPV-CPN categories.

Figure 7 visualizes the yaw and pitch head orientation of the manual SRTs and DHTs relative to all four categorization groups of the BPPV-CPN (true positive, false positive, true negative, and false negative). For all four categories, the general distribution seemed to be randomly scattered with no apparent pattern, except for the more accurate left SRT yaw-axis head angle in the true positive BPPV-CPN group (in line with the described findings in Table 4). This indicates no clear relationship between the applied yaw- and pitch head angles and the BPPV-CPN categories.

### 3.2 Head movement (angular velocity and duration of movement) in the manual SRT and DHT

The TMDs duration of movements were for the SRTs performed within the targeted 2-s [supine position to the right SRT: 1.4 s (95% CI: 1.4–1.4); the right SRT to the left SRT: 1.8 s (95% CI: 1.8–1.9). The duration of movement with the DHTs was slightly above 2 sec upright to the right DHT supine position: 2.1 (95% CI: 2.0–2.1); upright to the left DHT supine position: 2.1 (95% CI: 2.0–2.2)] (Table 5).

**Table 5:** Head movement and BPPV-characteristic positional nystagmus with traditional manual diagnostics (*n* = 198).

	Total		BPPV-CPN		No BPPV-CPN		*p*-value
	Mean	(95% CI)	Mean	(95% CI)	Mean	(95% CI)	
Supine Roll test							
*From the supine position to the right side*	*n* = 192		*n* = 17		*n* = 175		
Mean velocity, °/s	34.0	(32.6, 35.4)	38.8	(31.4, 46.1)	33.5	(32.2, 34.9)	0.156
Peak velocity, °/s	144.4	(137.8, 151.0)	147.6	(120.0, 175.1)	144.1	(137.2, 151.0)	0.794
Duration of movement, s	1.4	(1.4, 1.4)	1.4	(1.3, 1.4)	1.4	(1.4, 1.4)	0.120
*From the right side to the left side*	*n* = 192		*n* = 21		*n* = 171		
Mean velocity, °/s	67.9	(69.8, 65.9)	71.0	(64.1, 77.8)	67.5	(65.4, 69.5)	0.342
Peak velocity, °/s	217.6	(208.6, 226.5)	211.8	(182.2, 241.4)	218.3	(208.8, 227.8)	0.658
Duration of movement, s	1.8	(1.8, 1.9)	1.8	(1.7, 1.9)	1.8	(1.8, 1.9)	0.745
Right Dix-Hallpike test							
*From the upright to the supine position*	*n* = 195		*n* = 47		*n* = 148		
Mean velocity, °/s	46.3	(44.9, 47.7)	47.2	(44.2, 50.2)	46.0	(44.5, 47.5)	0.494
Peak velocity, °/s	133.4	(127.9, 139.0)	138.3	(127.5, 149.1)	132.0	(125.6, 138.5)	0.355
Duration of movement, s	2.1	(2.0, 2.1)	2.1	(1.9, 2.2)	2.1	(2.0, 2.1)	0.966
Left Dix-Hallpike test							
*From the upright to the supine position*	*n* = 193		*n* = 30		*n* = 163		
Mean velocity, °/s	45.8	(44.4, 47.3)	46.7	(44.0, 49.3)	45.7	(44.0, 47.4)	0.524
Peak velocity, °/s	121.9	(117.2, 126.5)	118.1	(105.9, 130.2)	122.6	(117.5, 127.6)	0.495
Duration of movement, s	2.1	(2.0, 2.2)	2.0	(1.9, 2.1)	2.1	(2.1, 2.2)	0.134

BPPV-CPN: BPPV-characteristic positional nystagmus; TMD: traditional manual diagnostics. All p-values are obtained by unpaired t-test (Welch’s test: unequal variance). A *p*-value < 0.05 is considered significant (*). Please note that there were no statistically significant intergroup differences in angular velocity and duration of movement between groups with and without observed BPPV-CPN with TMDs.

**Figure 8:**
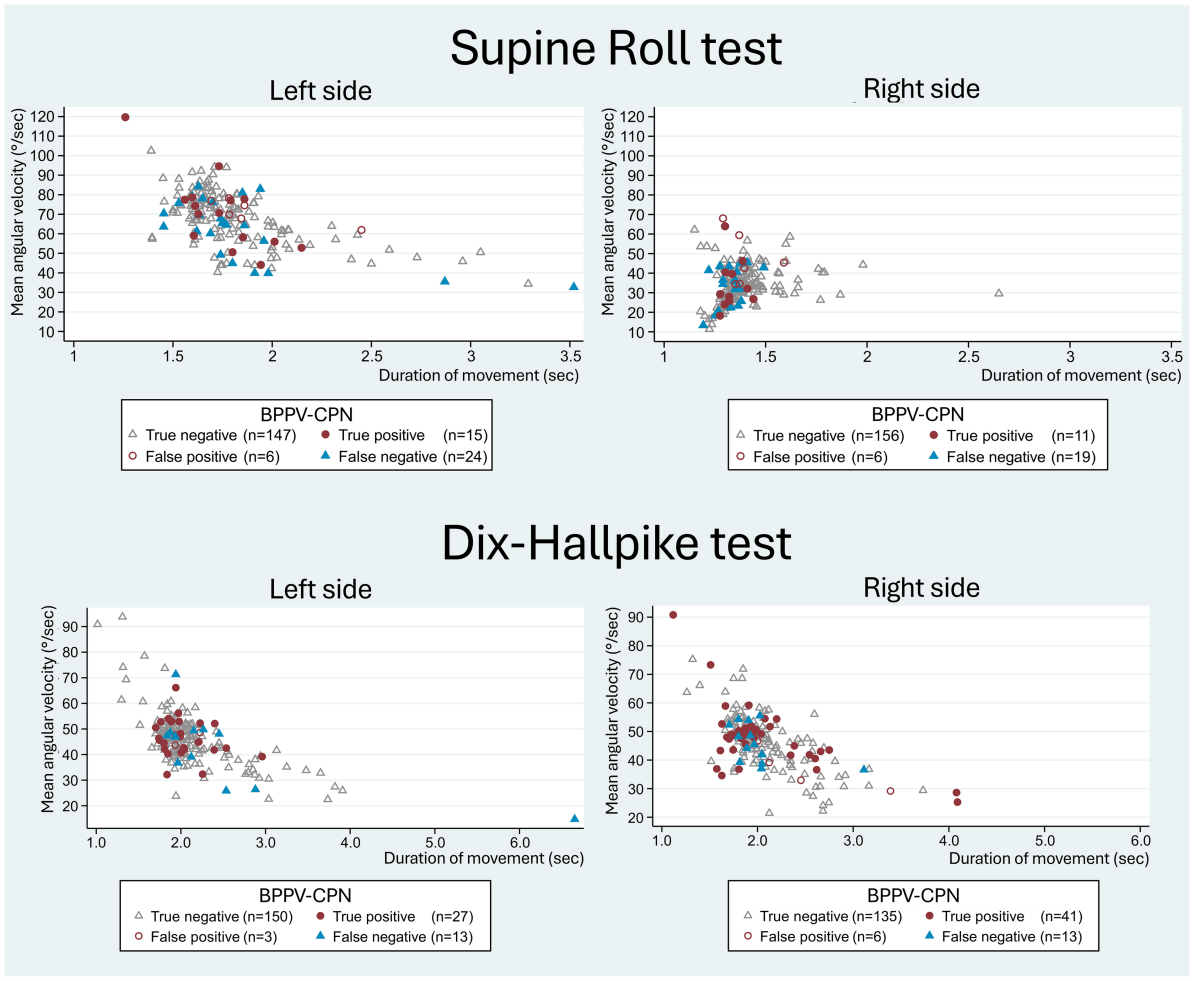
Head movement (mean angular velocity and duration of movement) with traditional manual diagnostics in relation to BPPV-characteristic positional nystagmus classification. The scatterplots show the duration of movement (x-axis) and the mean angular velocity (y-axis) of the head movement with the manual Supine Roll test (SRT) and manual Dix-Hallpike test (DHT), with symbols indicating the categories of BPPV-characteristic positional nystagmus (BPPV-CPN): true positive (red circle), false negative (blue triangle), false positive (red hollow circle), and true negative (grey hollow circle). The reference (true/false) was defined by the outcome of the corresponding test with mechanical rotation chair diagnostics (gold standard). With the SRT, the left scatterplot illustrates the 90° yaw-axis head rotation from the supine position to the right SRT, while the right scatterplot illustrates the 180° yaw-axis head rotation from the right SRT to the left SRT test position. With the DHT, both scatterplots represent the head movement from the upright position to the supine position. Please note that the data points for both the SRT and DHT appear randomly distributed without systematic differences between the BPPV-CPN classification groups.

A trend of a higher mean and peak angular velocity (except for the left DHT peak velocity) was observed in the group with detected BPPV-CPN during TMDs (Table 5). However, no significant difference in the angular velocities or duration of movement was found between the true positive and false negative groups (Table 6). Figure 8 illustrates this observation, displaying a random distribution of the angular velocity and duration of movement according to the categorization of the BPPV-CPN (true positive, false positive, true negative, and false negative). The majority of the SRTs and DHTs were performed within a narrow range of mean velocity and duration of movement (with few outliers), making it challenging to determine their individual or combined impact on the diagnostic test’s ability to reproduce the BPPV-CPN observed with MRC diagnostics.

**Table 6:** Head movement and true positive or false negative BPPV-characteristic positional nystagmus with traditional manual diagnostics (*n* = 198).

	True positive BPPV-CPN		False negative BPPV-CPN		*p*-value
	Mean	(95% CI)	Mean	(95% CI)	
Supine Roll test (*n* = 192)					
*From the supine position to the right side*	*n* = 11		*n* = 19		
Mean velocity, °/s	34.0	(25.4, 42.7)	33.3	(28.5, 38.2)	0.867
Peak velocity, °/s	145.8	(109.8, 181.9)	149.7	(117.9, 181.5)	0.868
Duration of movement, s	1.3	(1.3, 1.4)	1.3	(1.3, 1.4)	0.841
*From the right side to the left side*	*n* = 15		*n* = 24		
Mean velocity, °/s	70.7	(60.1, 81.3)	62.9	(56.4, 69.4)	0.166
Peak velocity, °/s	211.2	(170.3, 252.2)	215.9	(183.0, 248.8)	0.851
Duration of movement, s	1.7	(1.6, 1.9)	1.8	(1.7, 2.0)	0.322
Right Dix-Hallpike test (*n* = 195)					
*From the upright to the supine position*	*n* = 41		*n* = 13		
Mean velocity, °/s	48.1	(44.9, 51.2)	45.8	(41.7, 50.0)	0.393
Peak velocity, °/s	142.4	(131.6, 153.1)	129.3	(106.1, 152.6)	0.232
Duration of movement, s	2.1	(1.9, 2.2)	2.0	(1.8, 2.2)	0.742
Left Dix-Hallpike test (*n* = 193)					
*From the upright to the supine position*	*n* = 27		*n* = 13		
Mean velocity, °/s	46.7	(43.8, 49.6)	42.4	(33.8, 51.0)	0.321
Peak velocity, °/s	119.2	(105.5, 133.0)	122.0	(93.8, 150.2)	0.831
Duration of movement, s	2.0	(1.9, 2.1)	2.5	(1.9, 3.2)	0.060

BPPV-CPN: BPPV-characteristic positional nystagmus; TMD: traditional manual diagnostics. True and false BPPV-CPN are determined by the BPPV-CPN observed during diagnostics with the mechanical rotation chair (gold standard). All p-values are obtained by unpaired t-test (Welch’s test: unequal variance). A *p*-value < 0.05 is considered significant (*). Please note that there were no statistically significant intergroup differences in velocity and duration of movement between the groups with true positive and false negative BPPV-CPN with TMDs.

## 4 Discussion

### 4.1 Key findings

With this study, we aimed to investigate whether head orientation and -movement (angular velocity and duration of movement), measured during the TMD (SRT and DHT), might affect the outcome of BPPV diagnostics when compared to MRC diagnostics with fixed positions (the gold standard).

#### 4.1.1 Head orientation in the manual SRT

Significant head angle inaccuracies (relative to the target head angle) were observed for all head angles in the right and left SRT (Table 3). These inaccuracies were predominantly seen with the SRT yaw-axis head angles (right SRT: 70.3° (95% CI: 68.7, 71.9); Left SRT: −66.2° (95% CI: −67.7, −64.6)), which were considerably undershot in relation to the target head angle of ± 90°. The manually carried out SRT missed the identification of BPPV-CPN in more than 60% of patients (false negatives in the right SRT: 63.3% (19/30); false negatives in the left SRT: 62.5% (25/40)) (Supplementary Table C1). However, there were no significant differences in the mean head angles when comparing true positives and false negatives, except for the left SRT yaw-axis head angle. For the left SRT yaw head angle, the true positive group demonstrated significantly greater accuracy than the false negative (true positive: −69.8° (95% CI: −77.6, −61.9); false negative: −60.5° (95% CI: −65.5, −55.5); *p* = 0.034) (Table 4). While this finding supports the hypothesis that precise head orientation improves diagnostic performance, this association must be highlighted as weak, as the same pattern was not observed for the remaining SRT yaw- and pitch-axes head angles.

The false negative group with SRTs exhibited an overall higher variability (a greater spread in the applied head angles) in the yaw and pitch head angles compared to the true positive group (Figure 6). A scatterplot combining the right- and left-sided SRT data suggests potential cutoff values of the head angles, where the chance for a correct diagnostic outcome is best. The distribution in this scatterplot indicates that the minimum SRT yaw head angle required for eliciting BPPV-CPN might be approximately ± 55° (right vs. left SRT). The minimum SRT pitch head angle required for eliciting BPPV-CPN may be between −60 ° and −75°, suggesting that it is preferable to apply a pitch head angle beyond the −60° target rather than not reaching it. However, these suggested minimal head angles required for eliciting BPPV-CPN should be interpreted cautiously as weak tendencies due to small subgroups sizes and the exploratory nature of the results. When combining both the SRT yaw- and pitch head angle in the same scatterplot, a more random distribution of the true positive, false positive, true negative, and false negative groups is seen (Figure 7) as compared to the discussed individual scatterplots of the SRT yaw- and pitch head angles (Figure 6). To validate such threshold head angles, future studies should consider applying logistic regression models or Receiver Operating Characteristic (ROC) analysis to larger datasets.

As previously mentioned, the SRTs failed to elicit BPPV-CPN in a substantial proportion of participants (false negatives). This lower ability to induce BPPV-CPN during the SRT might be explained by the fact that most participants were unable to manually reach the ±90° target yaw-axis head rotation, which was imposed using MRC diagnostics due to the fixed positions. The observed difficulty with reaching the target SRT yaw head angle confirms previous findings indicating that even with young, healthy individuals, it might not be possible to achieve a target head angle of ± 90° with only cervical rotation (50). This highlights and supports the potential benefits (and necessity) of incorporating a mandatory full-body rotation or additional upper trunk rotation with the manual SRT to optimize the detection of lateral canal BPPV. Performing a full-body rotation may prove challenging due to the dimensions of a standard examination bed, suggesting that additional trunk rotation to reach the 90° target angle might be a more feasible option in daily practice.

#### 4.1.2 Head orientation in the manual DHT

Significant head angle inaccuracies (relative to the target head angle) were observed for all head angles in the right and left DHT (Table 3). Compared to the SRT, the DHT yaw head angles were overall closer to the target head angle (± 45°), with the right DHT being notably more accurate than the left DHT [right DHT: 47.4° (95% CI: 46.2, 48.7), left DHT: −33.3° (95% CI: −34.6, −31,9)]. This difference in accuracy between the right and left DHT yaw head angle might be influenced by the examiner’s dominant hand (right-handed). However, our finding of higher accuracy of the yaw head angle on the ipsilateral side of the dominating hand contradicts the findings of a previous study that described a higher accuracy of the yaw head angle on the contralateral side of the dominating hand (when performing the Epley maneuver) (51). Despite the inaccuracies in the DHT positions, the manual DHT correctly identified the BPPV-CPN in most patients when compared with the MRC diagnostics [true positive in right DHT: 73.2% (41/56); true positive in left DHT: 65.9% (27/41)] (Supplementary Table C1).

True BPPV-CPN seemed to be more frequently identified when the DHT yaw head angle was beyond the target ±45° (more true positives) (Figure 6). Conversely, for DHT pitch head angle, true positives were more frequent when the pitch head angle did not reach the target −120° (between −100° and −120°), indicating that reliable DHT results might be achievable even with limited cervical mobility. However, as mentioned above, this should be interpreted cautiously as weak tendencies due to small subgroup sizes and the exploratory approach. When combining both the yaw- and pitch head angles in the same scatterplot, a more random distribution of the true positive, false positive, true negative, and false negative groups was seen (Figure 7) as compared to the discussed individual scatterplots of the yaw- and pitch head angles (Figure 6).

#### 4.1.3 Head movements (angular velocity and duration of movement) in the manual SRT and DHT

While this study revealed substantial variation in the angular velocity and duration of movement with TMD (Figure 8), the majority of movements occurred within a quite narrow range of velocities (Table 5), making it challenging to identify specific cutoff values where the probability of a true positive outcome is optimal. Furthermore, no significant differences in the angular velocities and duration of movements were identified between the true positive and false negative groups (Table 6).

We observed a significant difference in the yaw axis head angle for the left SRT between the groups with and without observed BPPV-CPN. This difference was not observed for the right SRT. This difference might be caused by the SRT being performed with a continuous rotation from the right to the left side. This affected the velocities of the head movements to each side, which may have affected the ability to produce a BPPV-CPN, which is acknowledged as a study limitation.

### 4.2 Comparison with existing literature

To the best of our knowledge, this study is the first to analyze the impact of the head orientation and -movement on the outcome of TMD. Therefore, direct comparison with other studies is not possible. Instead, we discuss our findings in relation to existing theories and research dealing with BPPV diagnostics.

The main finding of this study was unexpected, as we found no significant difference in imposed head orientation and -movements between the groups that successfully identified BPPV-CPN (true positive) and those who did not (false negative). This finding could partly be explained by the inherent challenges associated with BPPV diagnostics, particularly the assumed heterogeneity of BPPV pathophysiology (otoconia size, quantity, localization, etc.) (36, 37). This heterogeneity might, at least theoretically, explain why the importance of the specific head orientation and -movement is subject to various degrees of inter-individual variability. Precise head orientation and minimum angular velocities might be critical when the otoconia are clustered within the SCC, with different optimal values depending on individual otoconia characteristics (36, 37). Conversely, with very dispersed otoconia, otoconial movement may occur as long as the head moves in a way that aligns the affected SCC with the gravity vector, regardless of the precise angulation of the head orientation. The latter assumption aligns with the minimum stimulus theory as proposed by Libonati et al. (52), which uses the upright BPPV protocol to elicit BPPV-CPN with minimal SCC stimulus (by minimal angulation in the head orientation), aiming to increase patient cooperation by minimizing the induced discomfort. This protocol includes a 30° lateral head bend bilaterally (rotation around the roll axis) for examination of the lateral SCCs. The vertical SCCs are examined in pairs (left anterior and right posterior; right anterior and left posterior) by rotating the patient’s head 45° to the right and left side, respectively, followed by a slow cervical flexion (30°) and extension (60°). If no nystagmus occurs in these minimum stimulus positions, the examination is followed by a TMD (52). The upright BPPV protocol was reported to show high BPPV identification rates in previous studies [lateral canal BPPV: 95.5% (compared to a diagnostic protocol including the upright BPPV protocol, supine position, and SRT); posterior canal BPPV: 87.2% (compared to DHT)] (53, 54). However, the observation that minimal head orientation is sufficient to identify the majority of lateral canal BPPV is contradicted by our findings. We observed the opposite: the manual SRT, failing to reach the target −90° yaw-axis rotation, was unable to identify 63% of the BPPV-CPN detected during the SRT with an MRC (reaching the target ±90° rotation). It might be that this comparison does not hold due to a difference in orientation of the lateral SCCs in the SRT and the upright BPPV protocol (46, 52).

BPPV diagnostic outcomes are likely not solely determined by the accuracy of head orientation. Some participants showed BPPV-CPN during the TMDs but not with the MRC diagnostics (classified as false positives) (right SRT: *n* = 6; left SRT: *n* = 6; right DHT: *n* = 3; left DHT: *n* = 6) (Supplementary Table C1). This raises the hypothesis that the SRT’s and DHT’s reproducibility of the BPPV-CPN cannot reach 100%. This could, theoretically, be explained by the fact that free-floating displaced otoconia move with every diagnostic test procedure. It is doubtful that these otoconia will return to their exact initial position, and this will, per se, limit the reproducibility of the BPPV diagnostics. If it is true that BPPV may be overlooked despite accurate head orientation, it might turn out advantageous to repeat the BPPV diagnostic procedures in case you encounter a patient with a typical BPPV case history but with a negative diagnostic outcome (55).

With the head movements (angular velocity and duration of movement), we found no difference between the true positive and false negative BPPV-CPN with the TMDs.

In general, there exists a sparse amount of literature on the head movements in BPPV diagnostics. One biomechanical model suggests that inertial forces only play a minimal role in the movement of otoconia, potentially only disrupting the otoconia-wall interaction (37). A clinical study by Anurin et al. (56) found that DHT angular velocities between 100 and 200°/second triggered a more intense BPPV-CPN (higher average slow-phase velocity), suggesting that a high angular velocity increased the possibility of identifying a BPPV-CPN response. However, they did not report the same association with the SRT.

Given the fact that the MRC has a different angular velocity profile (the head is more excentric, resulting in stronger linear accelerations and a longer duration of movement) compared to the profile seen with TMDs, we cannot rule out that head movement variables might have caused, or at least to some extent contributed, to the higher sensitivity of BPPV-CPN identification as seen with the MRC diagnostics compared to the TMD. Anyway, the head movement profiles of TMD and MRC diagnostics differ.

Finally, the identification of BPPV-CPN and, hence, the diagnostic outcome (of both TMD and MRC diagnostics) is also highly dependent on the examiner’s identification and interpretation of the positional nystagmus. With many commercially available eye-tracking systems, accurate 3D detection and quantification of eye movements remains a significant challenge. Therefore, identification and interpretation of positional nystagmus remain subjective and, as a direct consequence thereof, highly dependent on the examiner’s level of experience. Accurate nystagmus observation with BPPV diagnostics requires specific and unambiguous patient instructions (eye position with a straightforward gaze and minimal blinking). Gaze direction affects the intensity and direction of nystagmus, especially in posterior canal BPPV. With posterior canal BPPV, the amplitude of the torsional component increases when the gaze is directed toward the affected ear, while the amplitude of the vertical component increases with the gaze directed toward the unaffected ear (57). With the interpretation of nystagmus, the risk of BPPV overdiagnosis is also possible, as positional nystagmus during BPPV diagnostics is commonly encountered (71–88%) in individuals without vertigo (38, 39). However, this type of asymptomatic positional nystagmus typically differs from BPPV-CPN by lacking a torsional component, having prolonged duration, and exhibiting a low average slow-phase velocity (38, 39). Assisted diagnostic tools, like deep learning models, have been suggested to improve the objectivity of the nystagmus interpretation and, thereby, reduce over- and/or misdiagnosis of BPPV (58, 59).

### 4.3 Strengths and limitations

A randomized controlled crossover design was chosen to minimize bias. A key strength was the focus on conducting the study in the same clinical context where the results are to be interpreted, with a study population carefully selected to reflect this context. However, despite the relatively large overall sample size (*n* = 198), some of the subgroups were inevitably rather small.

For all BPPV diagnostics (TMD and MRC diagnostics), we opted to execute this study with only one examiner, ensuring consistency in the performance of the diagnostic tests and the identification and interpretation of positional nystagmus. To validate the examiner’s performance in nystagmus identification and interpretation, a blinded expert reviewed all recorded eye videos. The agreement between these interpretations was overall satisfying, with no difference between the interpretation of posterior and non-posterior canal BPPV. However, the agreement tended to improve in the second study period, suggesting either bias from a learning effect (in nystagmus interpretation) or improved quality of the eye video recordings as the examiner gained more experience (34). We found, however, no indication that this potential learning effect influenced the imposed head orientation (Supplementary Table E). Conversely, the head movement of the TMDs did differ between the study periods, with significantly shorter duration of movements and higher angular velocities for the majority of the head movements in the second study period (Supplementary Table F). The risk of a learning effect was further reinforced by the nature of the intervention of this study, where total blinding of the examiner was not possible. The impact of the lack of blinding was attempted to be minimized by allowing the examiner to assess head orientation by eye measure only (with no feedback). Additionally, the head orientation and -movement may have been influenced by physical constraints of the participants (e.g., neck mobility or high BMI) and should be acknowledged as a possible limitation to this study.

The analysis of the association between the diagnostic outcome and the performance of the TMDs (head orientation and -movement) was restricted to the intra-examiner variability seen with that one examiner, introducing a risk of operator bias. Using several different examiners might have revealed a more nuanced pattern with the results. Another limitation in relation to the imposed head orientation and -movement is the 6DOF IMU’s attachment on top of the VNG goggles, making the VNG goggles’ position a proxy measure of the orientation of the head. The risk of measurement error by slippage of the VNG goggles was minimized by careful manual fixation throughout the procedure. Data with movement artifacts was excluded. Ideally, the 6DOF IMU-related uncertainty could be avoided by fixating it on the skull, i.e., with a bite board, which, however, is often not well tolerated by patients and less applicable in routine clinical practice.

Crossover studies are generally susceptible to carryover effects, and this study is no exception. Specifically, when considering the study protocol, the displaced otoconia were unlikely to remain in the exact location in the SCC(s) after the initial diagnostic test procedures (46). Therefore, the two diagnostic modalities did, most likely, not have identical starting points, introducing a potential carryover effect. This fundamental challenge was mitigated by (1) a washout period of a minimum of 30 min minimizing fatigue-related influence on the vertigo and the nystagmus (43) and (2) randomization of the order of the TMDs and MRC diagnostics. Furthermore, the randomization also reduced and minimized any confirmation bias identification and interpretation of positional nystagmus in the second test modality was influenced by the interpretation of positional nystagmus in the first test modality. The distribution between the observed BPPV-CPN with TMDs and MRC diagnostics seemed to be comparable between the two randomized groups, indicating that the positional nystagmus interpretation was not influenced by confirmation bias (Supplementary Tables C2, C3).

### 4.4 Impact and future research

The results of this study are applicable to adults undergoing TMD (with VNG goggles) when performed by a trained examiner. The generalizability of this study may be reduced, when applied to less experienced clinicians. This study showed that the inaccuracy of the manual DHT did not seem to influence its ability to identify most of the BPPVs. In contrast, the manual SRT with only head rotation often failed to achieve sufficient yaw head angles and simultaneously failed to identify the majority of the BPPV-CPN seen with lateral canal BPPV. This may lead to missed, incorrect, or delayed diagnosis in real-world clinical settings with the risk of psychosocial consequences and expanded healthcare costs. This challenge in diagnosing lateral canal BPPV might be overcome by modifying either the examiner training or the standard manual diagnostic procedure. A simple solution might be to apply head and upper trunk rotation, or a full-body roll to replace the head-only rotation in the SRT. An alternative approach to improving head orientation in the TMD is to utilize a guidance system.

As this study focused on the performance of TMDs in a clinical context, future research should employ a more rigorous and systematic approach to define the minimum head angles of head orientation and the optimal head movement profile (angular velocity and duration of movement) to identify BPPV-CPN during the SRT and DHT. Ideally, such a study would include patients diagnosed with BPPV and systematic rotation of their heads until BPPV-CPN is elicited.

Finally, this study used the BáránySociety diagnostic criteria (1) and did not address the presence of both positional vertigo and BPPV-CPN during the TMD. We highly recommend that future studies explore the relationship between positional vertigo and head orientation, as well as -movement. This information might add significant scientific information to internationally standardized BPPV diagnostic criteria.

## 5 Conclusion

This study examined the relationship between the diagnostic outcome of the traditional manual BPPV diagnostics and the imposed head orientation and -movement in traditional manual BPPV diagnostics (Supine Roll test and Dix-Hallpike test). Diagnostics with a mechanical rotation chair, where the target head orientations were fixed, serving as the gold standard. The results demonstrated that the inaccuracy of the imposed head orientation and -movements with the manual Dix-Hallpike test did not seem to influence the ability to identify posterior canal BPPV correctly. In contrast, the manual Supine Roll test (with only cervical rotation) consistently undershot yaw head angle and failed to identify a significant amount of lateral canal BPPV. We, therefore, recommend that the manual SRT should be performed with a full-body rotation or additional upper trunk rotation to achieve the 90° target yaw angle required to diagnose lateral canal BPPV.

In addition, we strongly encourage future research to employ a systematic approach to determine the specific and optimal head orientation and -movements for diagnosing BPPV, as well as to investigate the impact of the head orientation and -movements upon the various canalith repositioning maneuvers.

## Data Availability

The raw data supporting the conclusions of this article will be made available by the authors, without undue reservation.
