# Supplementary material for: Impact of head orientation and head movement in traditional manual diagnostics of benign paroxysmal positional vertigo: a randomized controlled crossover study
Source: Front Neurol. 2025 Oct 3;16:1654404. doi: 10.3389/fneur.2025.1654404 (PMC12531064; doi:10.3389/fneur.2025.1654404)
Supplement: Supplementary file 1 [file Data_Sheet_1.pdf]

Supplementary Material

<table>
<tr><th colspan="6">Table A. Comparison of included and excluded participants (n=279)</th></tr>
<tr><td></td><td colspan="2">Included (n=198)</td><td colspan="2">Excluded (n=81)</td><td>p-value</td></tr>
<tr><td>Sex:</td><td></td><td></td><td></td><td></td><td></td></tr>
<tr><td>Female, n (%)</td><td>139</td><td>(70.2)</td><td>52</td><td>(65.0)</td><td>0.397</td></tr>
<tr><td>Age, years (mean, ± SD)</td><td>58.6</td><td>15.7</td><td>59.1</td><td>15.7</td><td>0.809</td></tr>
<tr><td colspan="6">A p-value < 0.05 is considered significant (*). Please note that there were no significant differences in sex and age between the groups of included and excluded participants. The table is modified according to (31).</td></tr>
</table>

Table B. Head orientation in traditional manual diagnostics and true positive or false negative BPPV diagnosis (n=198)

	Target head angle	Imposed head orientation with TMD						p-value
		Total		True positive diagnostic conclusion		False negative diagnostic conclusion		
		Mean	(95% CI)	Mean	(95% CI)	Mean	(95% CI)	
**Supine Roll test**		n=192		n=8		n=17		
Supine position								
Yaw axis, °	0.0	1.7	(1.0, 2.4)	0.8	(-4.6, 6.1)	2.4	(-0.1, 5.0)	0.473
Pitch axis, °	-60.0	-56.7	(-58.1, -55.4)	-61.5	(-67.3, -55.8)	-56.0	(-62.6, -49.4)	0.267
Roll axis, °	0.0	-5.5	(-6.3, -4.7)	-7.6	(-15.4, 0.1)	-3.9	(-5.8, -2.0)	0.307
Right Side								
Yaw axis, °	90.0	70.3	(68.7, 71.9)	66.9	(61.0, 72.8)	67.8	(60.8, 74.9)	0.823
Pitch axis, °	-60.0	-64.4	(-65.8, -63.1)	-66.6	(-69.9, -63.3)	-63.8	(-70.4, -57.1)	0.419
Roll axis, °	0.0	-8.9	(-10.4, -7.4)	-2.6	(-14.2, 9.0)	-6.9	(-10.8, -3.1)	0.428
Left Side								
Yaw axis, °	-90.0	-66.2	(-67.7, -64.6)	-67.2	(-82.7, -51.7)	-61.4	(-68.2, -54.6)	0.382
Pitch axis, °	-60.0	-63.4	(-64.7, -62.2)	-63.9	(-70.0, -57.7)	-65.4	(-71.6, -59.3)	0.736
Roll axis, °	0.0	-2.3	(-4.0, -0.5)	-10.5	(-22.9, 1.9)	-1.3	(-7.3, 4.8)	0.217
**Right Dix-Hallpike test**		n=195		n=41		n=13		
Upright position								
Yaw axis, °	45.0	31.0	(30.0, 32.1)	30.7	(28.7, 32.8)	32.3	(26.2, 38.5)	0.502
Pitch axis, °	0.0	-0.3	(-1.0, 0.3)	-1.1	(-2.5, 0.4)	-1.4	(-4.7, 1.3)	0.803
Roll axis, °	0.0	-1.1	(-1.7, -0.5)	-0.6	(-1.8, 0.7)	-0.0	(-1.9, 1.9)	0.642
Supine position								
Yaw axis, °	45.0	47.4	(46.2, 48.7)	47.4	(44.8, 50.1)	45.3	(41.6, 49.0)	0.406
Pitch axis, °	-120.0	-112.2	(-113.8, -110.7)	-110.8	(-114.3, -107.2)	-111.0	(-118.3, -103.6)	0.958
Roll axis, °	0.0	3.8	(2.2, 5.4)	5.6	(2.3, 8.9)	2.8	(-4.2, 9.8)	0.444
**Left Dix-Hallpike test**		n=193		n=27		n=13		
Upright position								
Yaw axis, °	-45.0	-26.2	(-27.1, -25.2)	-26.0	(-28.4, -23.5)	-27.6	(-32.6, -22.6)	0.575
Pitch axis, °	0.0	1.1	(0.4, 1.8)	1.4	(-0.8, 3.7)	-0.7	(-3.6, 2.1)	0.304
Roll axis, °	0.0	-0.3	(-0.8, 0.2)	-1.0	(-2.4, 0.4)	0.5	(-2.1, 3.1)	0.244
Supine position								
Yaw axis, °	-45.0	-33.3	(-34.6, -31.9)	-34.8	(-38.5, -31.2)	-33.3	(-41.6, -25.1)	0.748
Pitch axis, °	-120.0	-111.3	(-112.8, -109.8)	-109.3	(-113.0, -105.6)	-116.2	(-123.5, -108.9)	0.055
Roll axis, °	0.0	-8.2	(-9.7, 6.7)	-9.9	(-12.4, -7.3)	-9.2	(-17.0, -1.4)	0.868

TMD: traditional manual diagnostics. True and false diagnostic conclusion is determined by the diagnostic conclusion from the mechanical rotation chair diagnostics (gold standard). Please refer to Table 1 for an overview of the BPPV-characteristic positional nystagmus required for a positive diagnosis in the Supine Roll test and Dix-Hallpike test. All p-values are obtained by unpaired t-test (Welch's test: unequal variance). A p-value < 0.05 is considered significant (*). The numbers from this table are used in Figures 5, 6, and 7. Please note that there were no statistically significant intergroup head angle differences between the groups with true positive and false negative diagnostic conclusions with TMD.

Table C1. BPPV-characteristic positional nystagmus in traditional manual diagnostics and mechanical rotation chair diagnostics

Total study population (n=198)

Right Supine Roll test

		**MRC**			
		BPPV-CPN	No BPPV-CPN	Total	p-value
**TMD**	BPPV-CPN	11	6	17	0.000*
	No BPPV-CPN	19	162	181	
	Total	30	168	198	

Left Supine Roll test

		**MRC**			
		BPPV-CPN	No BPPV-CPN	Total	p-value
**TMD**	BPPV-CPN	15	6	21	0.000*
	No BPPV-CPN	25	152	177	
	Total	40	158	198	

Right Dix-Hallpike test

		**MRC**			
		BPPV-CPN	No BPPV-CPN	Total	p-value
**TMD**	BPPV-CPN	41	6	47	0.000*
	No BPPV-CPN	13	138	151	
	Total	54	144	198	

Left Dix-Hallpike test

		**MRC**			
		BPPV-CPN	No BPPV-CPN	Total	p-value
**TMD**	BPPV-CPN	27	3	30	0.000*
	No BPPV-CPN	14	154	168	
	Total	41	157	198	

MRC: mechanical rotation chair; TMD: traditional manual diagnostics; BPPV-CPN: BPPV-characteristic positional nystagmus.
Results are expressed as absolute frequencies. P-values are obtained by Chi squared test (Fisher's exact test if expected cell counts are < 5). A p-value < 0.05 is considered significant (*). With BPPV detection, please note that the MRC diagnostics was more sensitive than the TMD for all subgroups.

Table C2. BPPV-characteristic positional nystagmus in traditional manual diagnostics and mechanical rotation chair diagnostics

Randomized to MRC diagnostics first (n=98)

Right Supine Roll test

		MRC			
		BPPV-CPN	No BPPV-CPN	Total	p-value
**TMD**	BPPV-CPN	4	4	8	0.010*
	No BPPV-CPN	9	81	90	
	Total	13	85	98	

Left Supine Roll test

		MRC			
		BPPV-CPN	No BPPV-CPN	Total	p-value
**TMD**	BPPV-CPN	6	5	11	0.003*
	No BPPV-CPN	11	76	87	
	Total	17	81	98	

Right Dix-Hallpike test

		MRC			
		BPPV-CPN	No BPPV-CPN	Total	p-value
**TMD**	BPPV-CPN	22	2	24	0.000*
	No BPPV-CPN	3	71	74	
	Total	25	73	98	

Left Dix-Hallpike test

		MRC			
		BPPV-CPN	No BPPV-CPN	Total	p-value
**TMD**	BPPV-CPN	15	2	17	0.000*
	No BPPV-CPN	3	78	81	
	Total	18	80	98	

MRC: mechanical rotation chair; TMD: traditional manual diagnostics; BPPV-CPN: BPPV-characteristic positional nystagmus.

Results are expressed as absolute frequencies. P-values are obtained by Chi squared test (Fisher's exact test if expected cell counts are < 5). A p-value < 0.05 is considered significant (*). For detection of BPPV, please note that MRC diagnostics was more sensitive than the TMD for all subgroups.

Table C3. BPPV-characteristic positional nystagmus in traditional manual diagnostics and mechanical rotation chair diagnostics

Randomized to TMD first (n=100)

Right Supine Roll test

		MRC			
		BPPV-CPN	No BPPV-CPN	Total	p-value
TMD	BPPV-CPN	7	2	9	0.000*
	No BPPV-CPN	10	81	91	
	Total	17	83	100	

Left Supine Roll test

		MRC			
		BPPV-CPN	No BPPV-CPN	Total	p-value
TMD	BPPV-CPN	9	1	10	0.000*
	No BPPV-CPN	14	76	90	
	Total	23	77	100	

Right Dix-Hallpike test

		MRC			
		BPPV-CPN	No BPPV-CPN	Total	p-value
TMD	BPPV-CPN	19	4	23	0.000*
	No BPPV-CPN	10	67	77	
	Total	29	71	100	

Left Dix-Hallpike test

		MRC			
		BPPV-CPN	No BPPV-CPN	Total	p-value
TMD	BPPV-CPN	76	1	77	0.000*
	No BPPV-CPN	11	12	23	
	Total	87	13	100	

MRC: mechanical rotation chair; TMD: traditional manual diagnostics; BPPV-CPN: BPPV-characteristic positional nystagmus

Results are expressed as absolute frequencies. P-values are obtained by Chi-squared test (Fisher's exact test if expected cell counts are < 5). A p-value < 0.05 is considered significant (*). With BPPV detection, please note that MRC diagnostics was more sensitive in than the TMD for all subgroups.

Table D. Traditional manual diagnostics ability to detect BPPV-characteristic positional nystagmus (n=198)

	Traditional manual diagnostics compared to mechanical rotation chair diagnostics									
	Sensitivity		Specificitity		PPV		NPV		Accuracy	
	%	(95% CI)	%	(95% CI)	%	(95% CI)	%	(95% CI)	%	(95% CI)
Right SRT	36.7	(19.9, 56.1)	96.4	(92.4, 98.7)	64.7	(38.3, 85.8)	89.5	(84.1, 93.6)	87.4	(82.8, 92.0)
Left SRT	37.5	(22.7, 54.2)	96.2	(91.9, 98.6)	71.4	(47.8, 88.7)	85.9	(79.9, 90.6)	84.3	(79.3, 89.4)
Right DHT	75.9	(62.4, 86.5)	95.8	(91.2, 98.5)	87.2	(74.3, 95.2)	91.4	(85.7, 95.3)	90.4	(86.3, 94.5)
Left DHT	65.9	(49.4, 79.9)	98.1	(94.5, 99.6)	90.0	(73.5, 97.9)	91.7	(86.4, 95.4)	91.4	(87.5, 95.3)

PPV: Positive predictive value; NPV: Negative predictive value; CI: Confidence interval; SRT: Supine Roll test; DHT: Dix-Hallpike test.

Table E. Head position in traditional manual diagnostics by study periods (n=198)						
	Target head angle	First Study Period (n=99)		Second Study Period (n=99)		p-value
		Mean	95% CI	Mean	95% CI	
**Supine Roll test**		(n=95)		(n=97)		
Right side						
Yaw axis, °	90.0	68.6	(66.3, 71.0)	72.0	(69.9, 74.0)	0.501
Pitch axis, °	-60.0	-64.5	(-62.3, -66.6)	-64.4	(-62.8, -66.0)	0.136
Left side						
Yaw axis, °	-90.0	-63.5	(-61.4, -65.7)	-68.8	(-66.8, -70.7)	0.068
Pitch axis, °	-60.0	-66.6	(-64.8, -68.5)	-60.3	(-58.9, -61.7)	0.647
**Right Dix-Hallpike test**		(n=98)		(n=97)		
Yaw axis, °	45.0	49.9	(48.0, 51.8)	45.0	(43.5, 46.5)	0.330
Pitch axis, °	-120.0	-114.3	(-111.9, -116.6)	-110.2	(-108.3, -112.1)	0.958
**Left Dix-Hallpike test**		(n=95)		(n=98)		
Yaw axis, °	-45.0	-36.7	(34.6, -38.8)	-29.9	(-28.4, -31.4)	0.732
Pitch axis, °	-120.0	-114.6	(-112.3, -116.9)	-108.1	(-106.4, -109.8)	0.085
All p-values were obtained with unpaired t-tests of the means between the groups defined by the first and second study periods (in case of unequal variance, Welch's t-test was used). A p-value is considered significant if p<0.05 (*). Please note that there was no significant difference between the head angle means when comparing the first and the second study periods.						

Table F. Head movement in traditional manual diagnostics by study periods (n=198)					
	First study period (n=99)		Second study period (n=99)		p-value
	Mean	(95% CI)	Mean	(95% CI)	
**Supine Roll test**					
From the supine position to the right side	n=95		n=97		
Mean velocity, °/s	33.0	(31.2, 34.8)	34.9	(33.0, 36.9)	0.157
Peak velocity, °/s	124.6	(115.3, 134.0)	160.4	(152.8, 168.0)	0.000***
Duration of movement, s	1.4	(1.4, 1.4)	1.4	(1.3, 1.4)	0.093
From the right side to the left side	n=95		n=97		
Mean velocity, °/s	61.8	(59.1, 64.5)	73.8	(71.5, 76.1)	0.000***
Peak velocity, °/s	181.4	(168.8, 194.1)	248.8	(240.1, 257.6)	0.000***
Duration of movement, s	1.9	(1.9, 2.0)	1.7	(1.6, 1.7)	0.000***
**Right Dix-Hallpike test**					
From the upright to the supine position	n=98		n=97		
Mean velocity, °/s	44.7	(42.4, 47.0)	48.0	(46.6, 49.4)	0.021*
Peak velocity, °/s	116.5	(108.0, 124.9)	148.8	(142.9, 154.6)	0.000***
Duration of movement, s	2.3	(2.2, 2.4)	1.9	(1.9, 1.9)	0.000***
**Left Dix-Hallpike test**					
From the upright to the supine position	n=95		n=98		
Mean velocity, °/s	43.7	(41.3, 46.1)	47.9	(46.3, 49.6)	0.005*
Peak velocity, °/s	109.0	(102.1, 116.0)	133.4	(128.2, 138.7)	0.000***
Duration of movement, s	2.3	(2.2, 2.5)	1.9	(1.9, 2.0)	0.000***
All p-values are obtained by unpaired t-test (Welch's test: unequal variance. A p-value < 0.05 is considered significant (*). A higher level of significance is marked in the following manner: p < 0.001 (**) and p < 0.0001 (***). Please note that every movement with traditional manual diagnostics, except for the movement from the supine position to the right Supine Roll test, was performed with a significantly higher angular velocity (mean and peak) and shorter duration of movement in the second study period compared to the first study period.					
